# Evaluating the locally sourced materials as fluid loss control additives in water-based drilling fluid

**DOI:** 10.1016/j.heliyon.2020.e04091

**Published:** 2020-05-30

**Authors:** Anietie N. Okon, Julius U. Akpabio, Kilaliba W. Tugwell

**Affiliations:** Department of Chemical and Petroleum Engineering, University of Uyo, Nigeria

**Keywords:** Chemical engineering, Locally sourced materials, Fluid loss control additive, Fluid loss volume, Filter cake thickness, Mud cake characteristics

## Abstract

In the exploration for hydrocarbons, a successful drilling operation to the desired depth hinges on the effective performance of the formulated drilling fluid. Apart from carrying drill cuttings to the surface, another major function of the fluid is to seal off the walls of the wellbore to prevent fluids from coming into and out of the wellbore while drilling a well. Numerous commercial fluid loss additives: carboxymethyl cellulose (CMC), polyanionic cellulose (PAC), among others have been in existence with their drawbacks and effect on the total drilling cost. This study evaluates the use of locally sourced materials: *Detarium microcarpum*, *Brachystegia eurycoma* and rice husk, as fluid loss control additive in the water-based drilling fluid. The materials were prepared, ground and sieved to 125 microns. Four sets of water-based drilling muds were formulated using the local materials and CMC as fluid loss control additives. The mud formulation was based on the American Petroleum Institute (API) standard of 25g bentonite to 350mL of water. Also, the filtration test of the formulated muds was performed using API recommended practice for static filtration test at low temperature - low pressure (LTLP) condition. The results obtained showed that *Detarium microcarpum* and rice husk fluid loss volume and filter cake thickness were comparable with that of CMC from additive content of 10g, while *Brachystegia eurycoma* was comparable from additive content of 15g. Furthermore, the composite additive results indicated that *Detarium microcarpum*-rice husk at 95% *Detarium microcarpum*-5% rice husk performed better than *Brachystegia eurycoma*-rice husk of the same combination. Additionally, the fluid loss volume and filter cake thickness of *Detarium microcarpum*-rice husk additive were comparable with CMC from 10g content. Also, the results revealed that the fluid loss volume and filter cake thickness obtained from the locally sourced materials were within API specification for fluid loss control agents. The mud filter cake characteristics exhibited by these materials depicted that they have slippery, smooth and soft mud cakes; thus, the characteristics of a good mud cake that will prevent differential pipe sticking.

## 1 Introduction

In the exploration for hydrocarbons, drilling a successful hole is an integral part of the process and is contingent upon the drilling fluid's performance (McCosh and Getliff, 2004 in Udoh et al., 2012). Udoh and Okon (2012) reported that drilling process involves the penetration of the earth's crust to several thousand feet where the hydrocarbons are accumulated in the reservoir using rotary drilling process to create a passage for the discovered hydrocarbon reserves to be produced at the surface. To achieve this cardinal objective of a drilling operation, the formulated drilling fluid used must exert its basic functions. In drilling engineering literature, drilling fluid is also referred to as “drilling mud”, and generally viewed as the “blood” of all drilling operations in the petroleum industry (Udoh et al., 2012). Annudeep (2012) maintained that drilling fluids are complex heterogeneous fluids, consisting of several additives used in the drilling of oil and natural gas wells since the early 1900s. According to Odunukwe (2015), a complete drilling fluid system must be properly designed to efficiently construct a well. Thus, some of the basic drilling fluid functions include removal of drill cuttings to the surface, bottom-hole cleaning, maintaining the wellbore stability, controlling high-pressure zones, etc. Among the enumerated drilling fluid functions, a major one is to seal the walls of the formation being drilled to prevent filtration. Hence, Feng et al. (2009) opined that one of the most desired properties of drilling fluid is the minimum fluid loss volume which can be achieved by the development of a low permeability filter cake on the wellbore. Therefore, every drilling fluid is designed to avoid a continuous fluid loss to the open-hole drilled which is highly undesirable (Azar and Samuel, 2007). Agwu and Akpabio (2018) added that drilling fluids are designed to reduce filtrate loss, form thin filter cakes that plaster the walls of the borehole to ensure minimal fluid loss and promote stability of the drilled well. As reported by Agwu et al. (2019), the continuous fluid loss to the formation led to thick mud cake which in some cases resulted in pipe sticking, drag and torque problems, among others. Besides, Kosynkin et al. (2011) mentioned that fluid invasion into porous media can damage reservoirs and reduce productivity by blocking hydrocarbon exit flow paths or causing wellbore collapse. Therefore, filtration control is important for both drilling performance and well productivity (Herzraft et al., 2001; Oleas et al., 2008).

In a drilling operation, filtration control is the addition of materials to drilling mud to reduce filtration rate and improve mud cake characteristics (Bourgoyne et al., 2003). The two mechanisms responsible for the filter cake build-up process during the drilling process are static and dynamic filtration mechanisms (Bageri et al., 2013). The static filtration occurs during drilling fluid non-circulation period, while dynamic filtration happens when the drilling fluid is in circulation. According to Agwu and Akpabio (2018), a thin filter cake has a low permeability which is an attractive property since it has a lot to do with the usability and functionality of the drilled wellbore, while a thick filter cake will cause lots of problems which include: differential pipe sticking, loss circulation, casing installation, etc. To handle this major functionality of drilling fluid, drilling mud engineers have been formulating drilling fluids with commercial polymers, namely, polyanionic cellulose (PAC) and carboxymethyl cellulose (CMC) as well as various polymers as fluid loss control additives (Caenn and Chillingar, 1996). However, Anawe-Paul and Adewale (2018) reported that these fluid loss control additives have limitations at high temperatures, high salinity or hardness and can also increase mud viscosity. They maintained that the commercial polymer CMC can withstand high temperature up to 300 °F, but deflocculates clay at low concentration, and lower gel strength and yield point of the drilling fluid. Also, sodium polyacrylate (PAC) is more temperature stable than CMC but it is extremely calcium sensitive. For starch as fluid loss control additive, it is relatively unaffected by water salinity or hardness, thus, mostly used in drilling fluids with high salt concentration but it cannot be used in drilling fluids that are exposed to high temperatures, as its thermal degradation begins at about 200 °F. Also, Bourgoyne et al. (2003) stated that starch is subjected to bacterial action and must be used with preservative except in salt-saturated water-based drilling fluids or drilling fluids with a pH above 11.5.

In recent times, there has been a shift in the use of commercial polymers to locally sourced materials as fluid loss control additives in water-based drilling fluids (Tugwell, 2018). According to Davoodi et al. (2018), the locally sourced additive has been introduced to the drilling industry for reducing the drilling fluid cost and the impact of the toxic chemicals on the ecosystem. In this direction, Agwu and Akpabio (2018) holistically looked at some of the works done in the use of local materials as fluid loss control additive in drilling fluids; as presented in Table 1. They mentioned that most studies were on water-based drilling fluid using sawdust, walnut shells, Arabic gum, starch (from cassava, corn and potato), rice husk, among others. Also, they pointed out that most of the researchers graduated the concentration of the material they used; most probably to know the optimal content of the material that would reduce filtration loss in drilling fluid. A look at these locally sourced materials used so far as fluid loss control additives in drilling fluids indicated that they are either cellulose (eg. sawdust, rice husk, groundnut husk, corn cob, walnut shell) or hydrocolloid (eg. gum arabic, starch, etc.) based products. For the hydrocolloid based products, they are very good thickeners, which is the potential of some of the locally sourced materials in this work. Hence, this study intends to evaluate two hydrocolloid based products: *Detarium microcarpum* and *Brachystegia eurycoma*, and one cellulose-based product, rice husk as fluid loss control additive in water-based drilling fluid to establish their filtration capabilities.

**Table 1:** Some studies results on filtration loss of some locally sourced materials.

Researcher(s)	Mud type	Agro waste used	Temperature & Pressure	Particle size range	Range of amount of agro waste used (g)	API filter loss (mL/30mins)
Bazarnova et al. (2001)	WBM	Carboxymethylated aspen wood (sawdust)	**-**	0.4–0.75mm	**-**	12–16
Iscan and Kok (2007)	WBM	Walnut shells	**-**	2–6mm	20–60	11–14.5
Hamida et al. (2010)	WBM	Waxy hull less barley	**-**	**-**	1–30	8 - 21 [unaged mud]; 3.9 [aged]
Hamida et al. (2010)	Saline mud	Waxy hull less barley	**-**	**-**	1–30	3–8.8
Olatunde et al. (2012)	WBM	Gum Arabic	150 °F; 100psi	**-**	32	17
Adebayo et al. (2012)	OBM	Sawdust	70 °C	1mm	-	8.6
Adebayo and Chinonyere (2012)	WBM	Sawdust	**-**	0.5–1mm	5–30	12–59
Egun and Abah (2013)	WBM	Cassava starch	**-**	**-**	2–8	4–8
Azizi et al. (2013)	WBM	Agarwood waste	**-**	45 and 90μm	6	13–16
Dagde and Nmegbu (2014)	WBM	groundnut husk	**-**	-	2–4	7.6 and 6.5
Okon et al. (2014)	WBM	Rice husk	**-**	125μm	5–20	16–42.5
Nmegbu and Bari-Agara (2014)	WBM	Corn cob cellulose	**-**	-	2–3	5.8 and 5.8
Anawe Paul et al. (2014)	OBM	Rice husk	60–100 °C	0.5 μm	5–25	137–171
Anawe Paul et al. (2014)	OBM	Sawdust	60–100 °C	0.5 μm	5–25	142–236
Anawe Paul et al. (2014)	OBM	Rice husk + Sawdust	60–100 °C	0.5 μm	5–25	178–234
Nyeche et al. (2015)	WBM	Potato starch	**-**	**-**	1–2	7–13.5
Ghazali et al. (2014)	WBM	Corn starch	170 - 200 °F	**<**125μm	6	31
Hossain and Wajheeuddin (2016)	WBM	Grass	**-**	35–300μm	0.25–1ppb	11–14.6
Samavati and Abdullah (2016)	WBM	*Ubi Kayu starch*	250 - 300 °F	-	14	0.4–250
Harry et al. (2016)	WBM	Cassava starch	80 °F	12–71μm	-	15–16
Chinwuba et al. (2016)	WBM	*Pleurotus tuber-regium*	Room temp. – 180 °F	-	5–6	8–10.8
Saengdee and Terakulsatit (2017)	WBM	Sugercane bagasse ash	25–80 °C	-	1–5% w/w	18–22.5

Source: Agwu and Akpabio (2018).

### 1.1 Overview of the locally sourced materials

The seeds of *Detarium microcarpum* and *Brachystegia eurycoma* in the local parlance are referred to as ‘Ofor’ and ‘Achi’ by the Igbos, and ‘Ogbogbo’ and ‘Akolodo’ by the Yorubas (Ikegwu et al., 2010). They belong to the same family of Leguminosae as well as the same sub-family of Caesalpinioideae, phylum of spermatophyte and order of Fabaceae (Nwosu, 2012). Additionally, they are dicotyledonous plants which commonly grow in the tropical rain forest of West Africa along river banks (Nwakaudu et al., 2017). According to Irondi et al. (2015), these plants are underutilized leguminous tree crops that have both food and medicinal uses. Uhegbu et al. (2009) added that these seeds' flour is used for thickening of traditional soups in the South-Eastern part of Nigeria. Abreu and Relva (2002) reported that the seeds are known to contain lipids, carbohydrates, protein, crude fibres and the essential elements, namely: Na, K, Mg, Ca, S, P and Fe. The element compositions in the seeds are presented in Table 2. On the other hand, paddy rice (*Oryza sativa*) is grown on every continent except in Antarctica, and the extent of paddy cultivation covers about one percent of the earth's surface (Esu, 2016). In Nigeria, rice cultivation is predominant in the following states: Kebbi, Niger, Taraba, Enugu, Ebonyi, Cross River, Akwa Ibom, Benue, Kaduna, etc. As reported by Prasad and SankhyanKarim (2000), paddy consists of about 72 percent of rice, 5–8 percent of bran, and 20–22 percent of husk. International Rice Research Institute (2016) mentioned that one kilogram (1kg) of rice results in about 0.28kg of rice husk as a by-product during the milling process. This agro by-product (rice husk) according to Akoko et al. (2012) and Kumar et al. (2012) is used in the field of civil engineering as concrete fibre and the electrical engineering field as insulating materials. Recently, studies have evaluated its substitute for fossil fuel to generate electricity from biomass process as a renewable energy source (Okon et al., 2014). Thus, rice husk contains 50% cellulose, 23–30% lignin, and 15–20% Silica (Ismail and Waliuddin, 1996; Ummah et al., 2015). Some of the elemental compositions of rice husk are presented in Table 2.

**Table 2:** Elemental compositions of the locally sourced materials.

	Elements composition	Locally sourced materials		
		*Detarium microcarpum*	*Brachystegia eurycoma*	Rice husk
i.	Silica	N/A	N/A	N/A
ii.	Alumina	N/A	N/A	0.01–0.025^b^
iii.	Ferric	N/A	N/A	0.005^b^
iv.	Calcium	0.34–0.35^a^	0.72–0.80^a^	0.01–0.02^b^
v.	Magnesium	0.10–0.17^a^	0.16–0.21^a^	0.005–0.02^b^
vi.	Sodium	0.13–0.14^a^	0.05–0.11^a^	0.002–0.005^b^
vii.	Potassium	0.15–0.18^a^	0.21–0.24^a^	0.002^b^
viii.	Phosphorus	0.15–0.18^a^	0.30–0.33^a^	N/A

Sources: a. Uhegbu et al. (2009); b. Subbukrishna et al. (2007).

## 2 Materials and methods

### 2.1 Samples preparation

**Figure 1:**
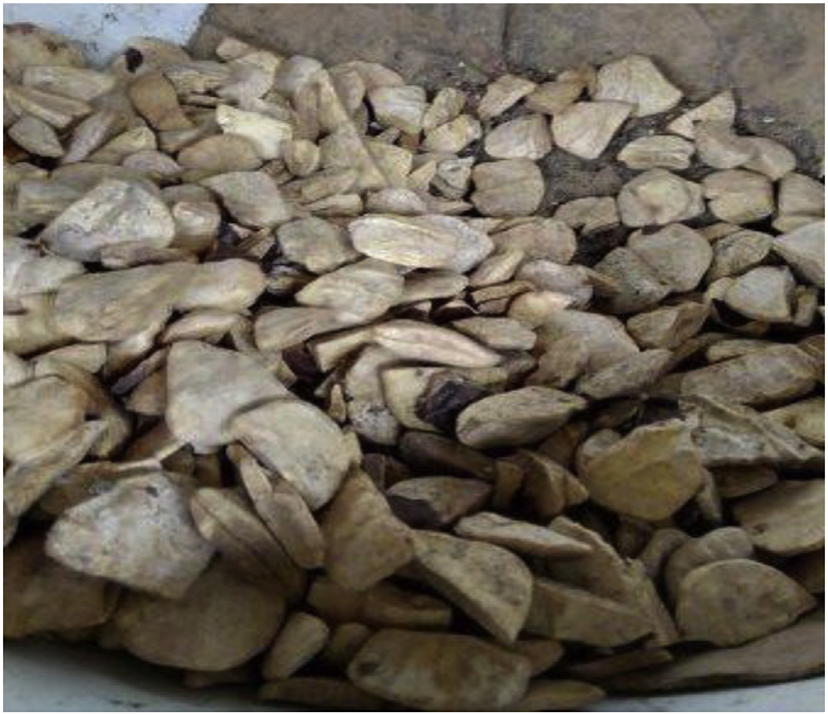
*Detarium microcarpum*.

**Figure 2:**
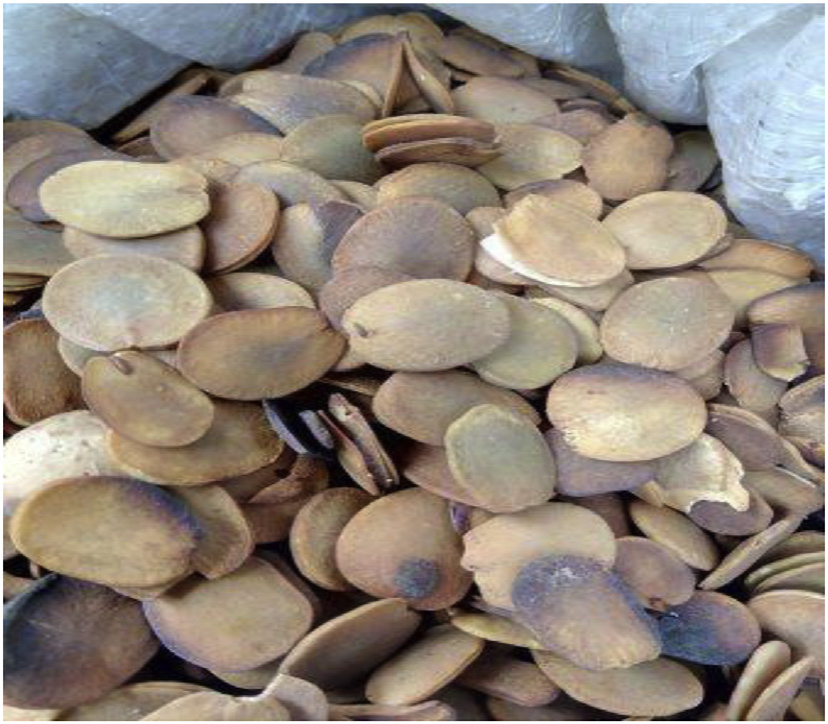
*Brachystegia eurycoma*.

**Figure 3:**
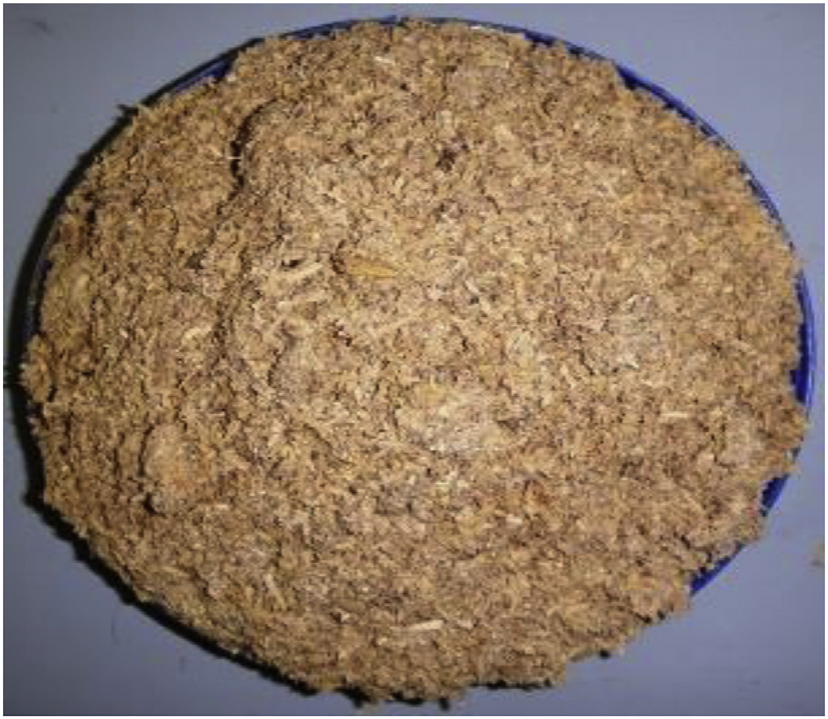
Rice husk.

**Figure 4:**
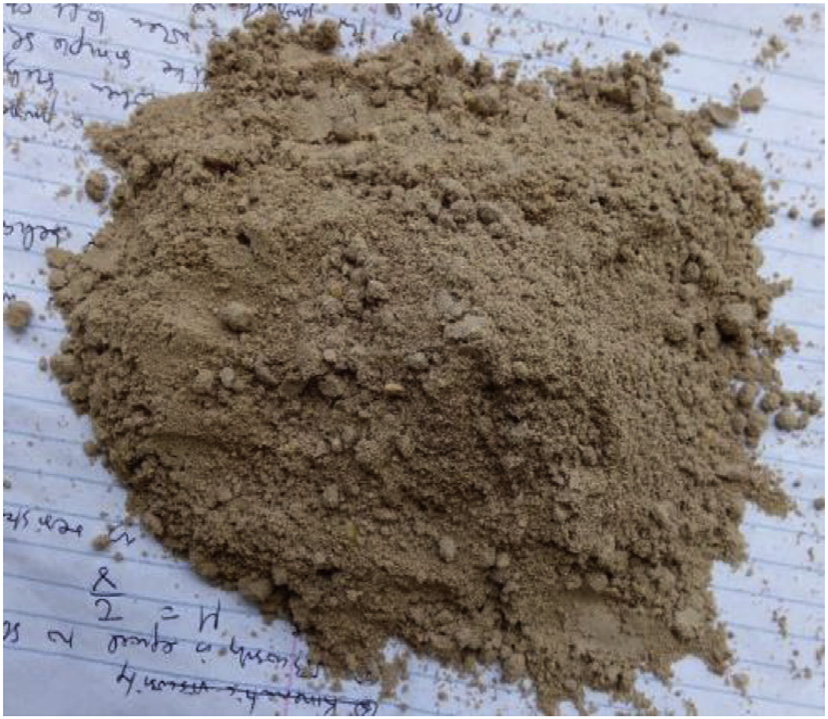
Ground *Detarium microcarpum*.

**Figure 5:**
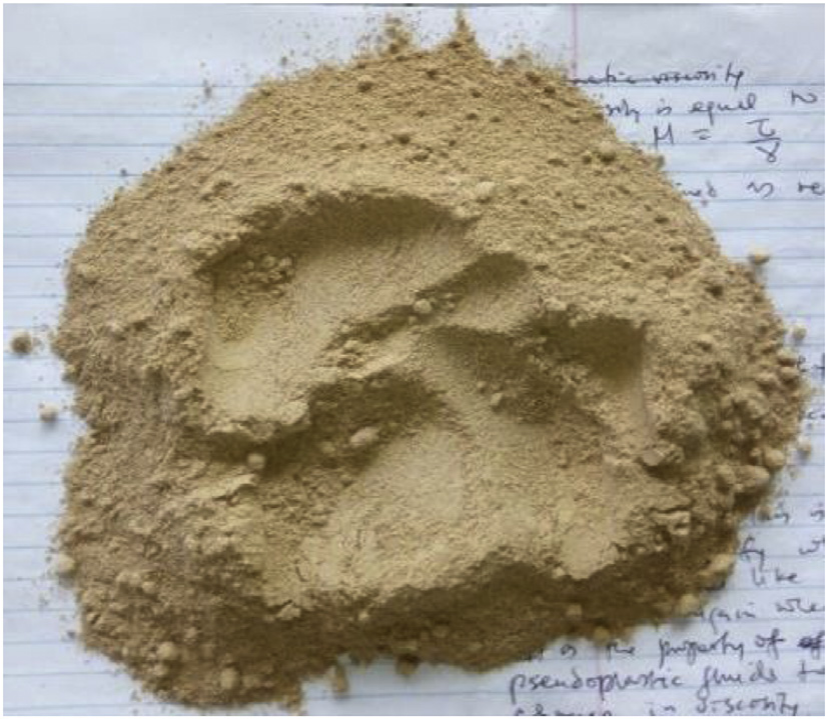
Ground *Brachystegia eurycoma*.

**Figure 6:**
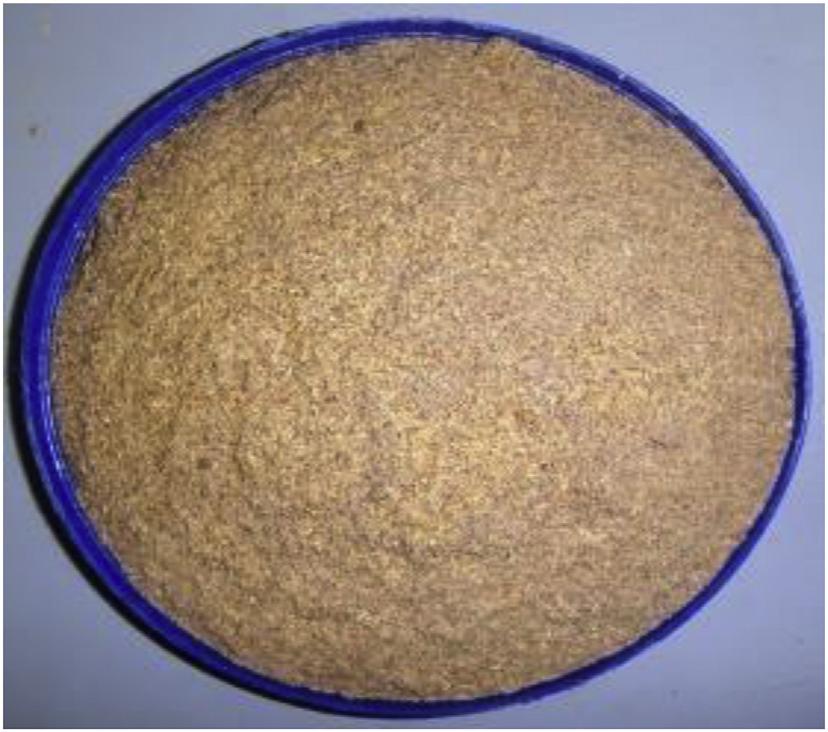
Ground *Rice husk*.

The seeds: *Detarium microcarpum* and *Brachystegia eurycoma* and rice husk (Figures 1, 2 and 3) used in this study were obtained from a local market and rice mill in Akwa Ibom State, Nigeria. The seeds were sorted to remove spoilt ones and then washed to remove debris. Afterwards, they were sun-dried for four (4) days and the cotyledons were extracted from the seeds (Figures 1 and 2) and soaked in distilled water. The water was decanted and the cotyledons were placed in a vacuum oven at a temperature of 70 °C to dry for 6 h. The dried cotyledons were ground using a blender and sieved to 125 μm to obtain fine textures (Figures 4 and 5). This particle size selection was based on the works of Ghazali et al. (2014) and Okon et al. (2014). The rice husk obtained from the mill (Figure 3) was spread on a sieve and its rice content was selected from the husk. Thereafter, the rice husk was placed in the vacuum oven for 5 h at a temperature of 40 °C to dry up any moisture content in it. The dried recipe (rice husk) was ground into small size with blender and sieved using sieve No. 125 μm to obtain fine particles as presented in Figure 6. These samples were stored in air-tight containers to avoid contamination.

### 2.2 Mud preparation

**Figure 7:**
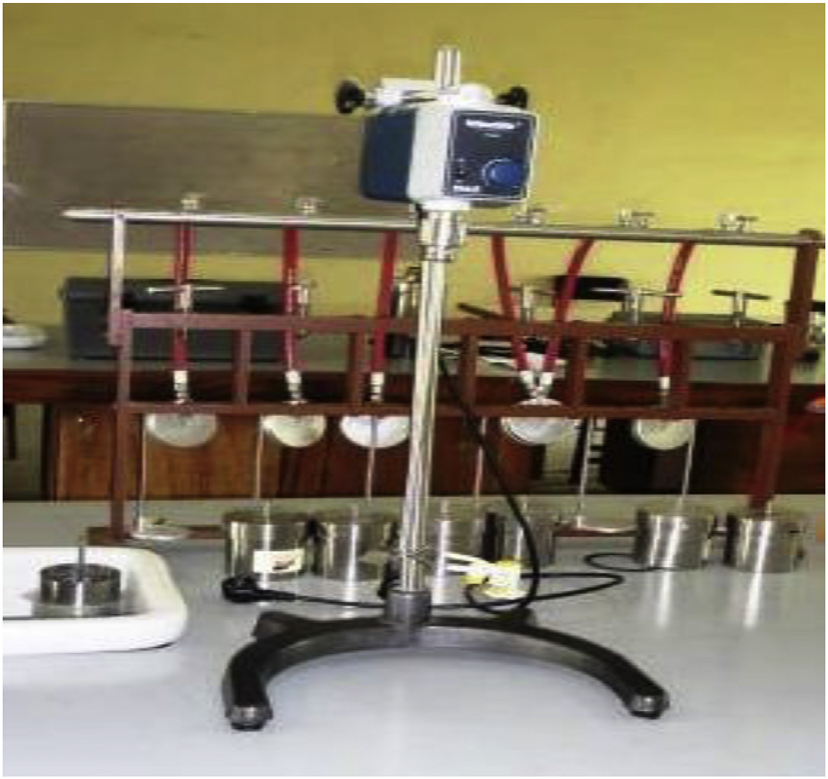
Hamilton Beach high-speed mixer.

**Figure 8:**
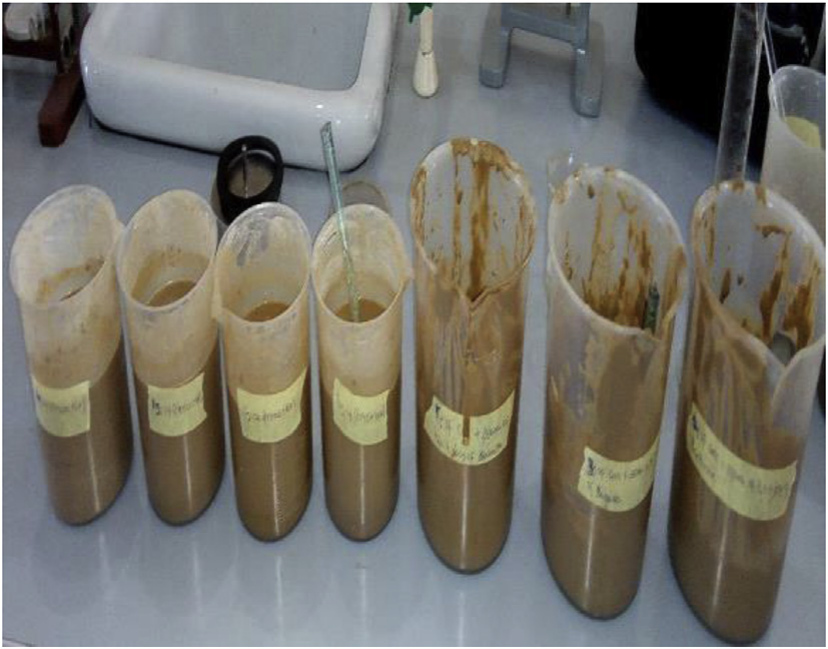
Some formulated drilling fluid samples.

Sixteen (16) drilling mud samples were prepared (formulated) based on the American Petroleum Institute (API) standard of 25g–350 mL of water for non-treated bentonite. Using Hamilton Beach Commercial high-speed mixer (Figure 7), the various components (additives) added to the distilled water to formulate the mud are presented in Table 3. These mud samples were grouped into four (4) sets, namely, SET-A through SET-D, with each set comprising of four (4) samples labelled 1 through 4. The group name (i.e., SET-A through SET-D) was based on the type of the fluid loss additive (*Detarium microcarpum*, *Brachystegia eurycoma*, rice husk and CMC) in the mud, while the mud sample number (e.g. SET-A1through SET-A4) depicts the fluid loss additive content (in gram) in the mud sample. Table 4 presents the various sample groups and their respective fluid loss additive content. For the CMC mud, since CMC is enhanced polymer (Okon et al., 2014), its content in the mud samples was not in the same proportion as the local additives. Furthermore, four (4) drilling mud samples with combined (composite) fluid loss additives were formulated. These mud samples fluid loss additives content were 95% *Detarium microcarpum* - 5% rice husk (MUD-A), 95% *Brachystegia eurycoma* - 5% rice husk (MUD-B), 5% *Detarium microcarpum* - 95% rice husk (MUD-C) and 5% a *Brachystegia eurycoma* - 95% rice husk (MUD-D). The formulated mud samples were kept to age for twenty-four (24) hours at room temperature. Some of these formulated drilling mud samples are shown in Figure 8. Thereafter, the filtration test of the mud samples was performed based on the API standard for the LTLP filtration test, and the fluid loss volumes and filter cake thickness of the mud samples were obtained and recorded.

**Table 3:** Drilling fluid basic compositions.

Mud components	Content	Mixing order	Mixing during (min)	Function
Distill water, (mL)	350	1	-	Base fluid
Bentonite, (g)	25	2	5	Viscosifier
Barite, (g)	10	3	5	Densifier
Soda ash, (g)	0.25	4	2	pH control
CMC, (g)	2, 4, 6, 8	5a	5	Fluid loss control additive
*Brachystegia eurycoma*	5, 10, 15, 20	5b	5	Fluid loss control additive
*Detarium microcarpum*	5, 10, 15, 20	5c	5	Fluid loss control additive
Rice Husk	5, 10, 15, 20	5d	5	Fluid loss control additive

**Table 4:** Drilling mud samples with their respective fluid loss additive content.

DM Mud (SET-A)		BE Mud (SET-B)		RH Mud (SET-C)		CMC Mud (SET-D)	
Name	Content (g)	Name	Content (g)	Name	Content (g)	Name	Content (g)
A1	5	B1	5	C1	5	D2	2
A2	10	B2	10	C2	10	D2	4
A3	15	B3	15	C3	15	D3	6
A4	20	B4	20	C4	20	D4	8

### 2.3 Mud filtration test procedure

**Figure 9:**
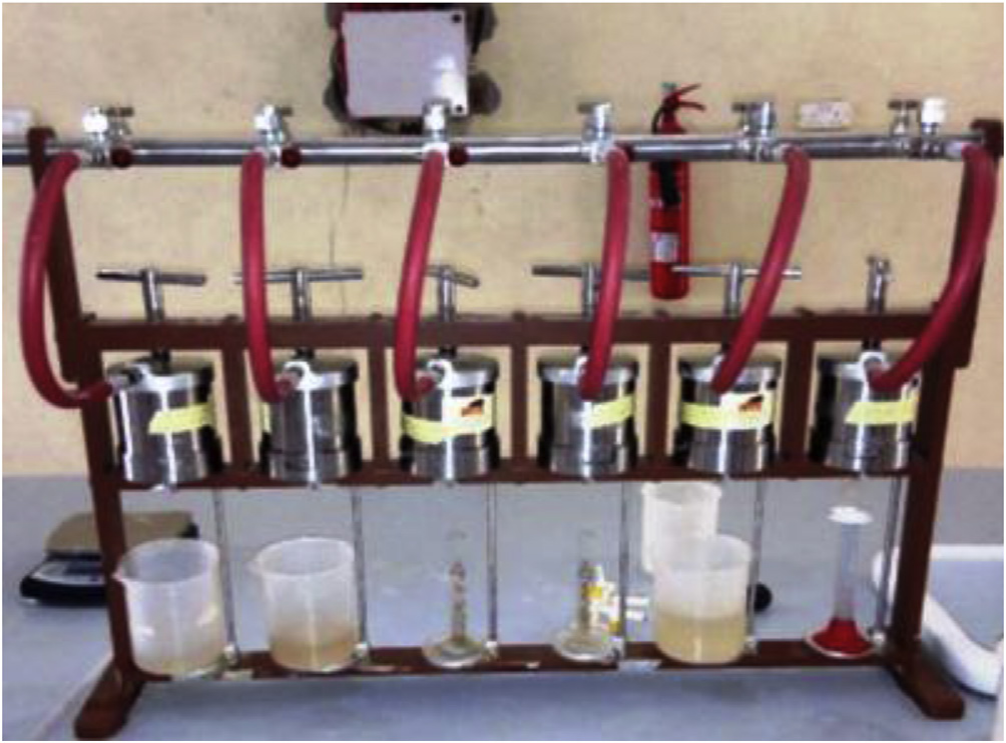
API LTLP filter press stack.

**Figure 10:**
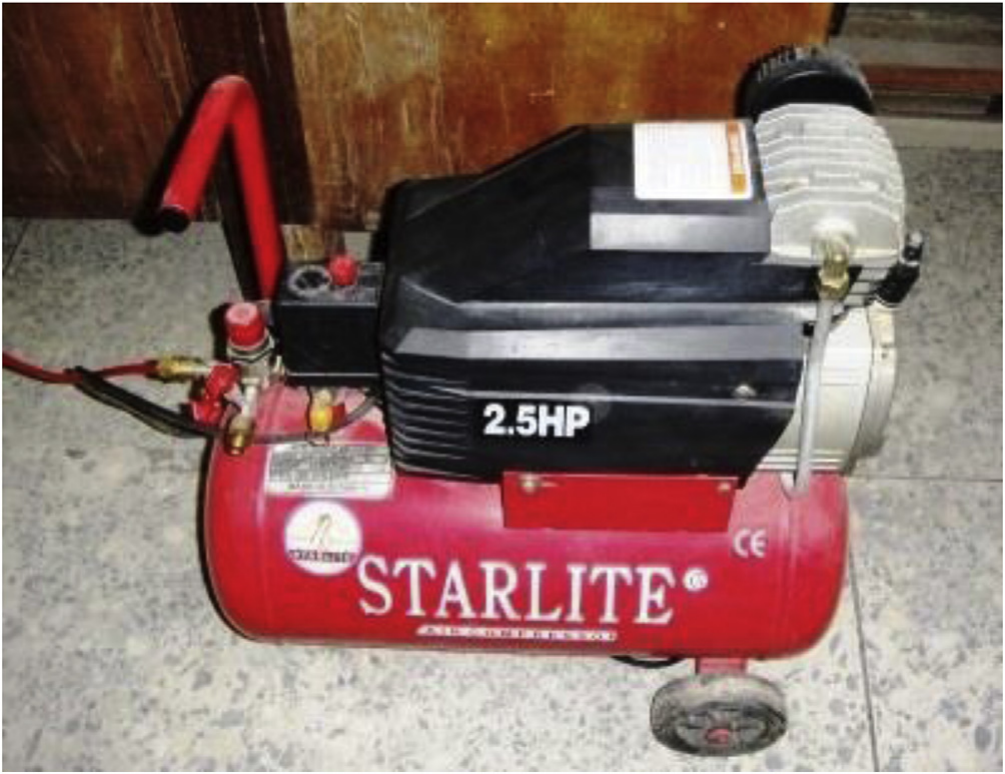
Air compressor pump.

The filtration test to evaluate the fluid loss control potential of the locally sourced material was based on the API recommended practice, API 13B-1. The standard recommends 100 psi (about 700 kPa) pressure and 30 min (i.e., 1800 s) for LTLP filtration test in the water-based drilling fluid. In this study, the LTLP filter press is a cylindrical cell of 3 inches internal diameter and 5 inches height to contain the drilling fluid samples. The API filter press consists of six (6) cylindrical cells on the stack (Figure 9). Whatman No. 50 papers were placed at the bottom of the cylindrical cells and the drilling fluid samples were poured into the cells. With the necessary connection in place, the recommended pressure was supplied from an air compressor pump (Figure 10) to the top of the cells. The filtrates from the drilling fluid samples were collected using measuring cylinders placed under the cells. In thirty (30) minutes, the volumes of the filtrate in the graduated cylinders were recorded in millilitres (mL) as the API fluid loss for the drilling fluid samples. Thereafter, cake thickness on the Whatman filter papers was measured with a digital vernier caliper and recorded as the drilling fluids’ cake thickness in millimetres (mm).

#### 2.3.1 Mud filter cake characteristic

Qualitative description of drilling fluid filter cake characteristics involves measurement of its thickness to establish whether the formed filter cake is either thick or thin. For the measurement of the qualitative characteristics of drilling fluid filter cake, Amanullah and Tan (2001) reported that there was no standard method to perform the evaluation rather it differs from researcher to researcher. Qualitatively, API describes drilling fluid filter cake as a firm, slippery, smooth, soft, sticky, etc (Agwu et al., 2019). Hence, from the appearance and texture of the formulated drilling fluid samples’ filter cakes, the qualitative characteristics of the mud cake were established based on API description.

#### 2.3.2 Mud filter cake permeability

The permeability of the mud filter cake controls both static and dynamic filtration (Rautela, 2000). It establishes the permissibility or transmissivity of the mud cake formed around the wellbore during the drilling operation. In other words, it depicts the ability of the formed mud cake to prevent or allow fluid (i.e., mud filtrate) to pass through it into the formation. Generally, the permeability of the filter cake is expressed as;

(1) $$k=q_{w}q_{c}\frac{\mu }{2t\Delta pA^{2}}$$

where;

k=mud cake permeability (Darcies, D)
$q_{w}=$mud filtrate (fluid loss) volume (cubic centimeter, cm^3^)
$q_{c}=$mud cake volume (cubic centimeter, cm^3^)
μ=mud filtrate viscosity (centipoise, cP)
t=time (seconds)
Δp=pressure differential (atmospheres)
$A^{2}=$filter cake area (centimeter, cm^3^)

Using conventional filter press data, Rautela (2000) presented Eq. (1) as;

(2) $$k=19.9q_{w}q_{c}\mu$$

where k is expressed in millidarcy (mD) and other parameters in Eq. (2) remains as expressed in Eq. (1). Regrettably, the drawback of Eqs. (1) and (2) is that the mud cake volume; $q_{c}$, is not a direct reflection of measurement derived from the filtration test experiment. Hence, Eq. (3) established by Lomba (2010) in Agwu et al. (2019) was used to evaluate the formulated mud samples' mud cake permeability. Thus;

(3) $$k=8.95\times 10^{-5}q_{w}\varepsilon \mu$$

where ε is the filter cake thickness and $q_{w}$ is the mud filtrate volume; all measured in millimetre (mm) while μ is as expressed in Eq. (2).

## 3 Results and discussion

The performance of the locally sourced materials as fluid loss control additives in water-based drilling fluid was evaluated based on the API specifications for standard polymer: CMC and PAC used in the industry. These specifications are presented in Table 5. The drilling fluid filtration properties evaluated to establish the potency of the locally sourced materials as fluid loss control additives were: filter loss volume, filter cake thickness, and mud cake permeability in non-composite and composite drilling mud samples.

**Table 5:** API specifications for fluid loss control additive.

	Fluid loss control additives	Filtration Test	
		Filter cake thickness	API Fluid loss
i.	Carboxymethyl cellulose (CMC)	>2mm	1.0 × 10^−5^m^3^ (10mL) max.
ii.	Polyanionic cellulose (PAC)		2.5 × 10^−5^m^3^ (25mL) max.

Source: Agwu et al. (2019).

### 3.1 Fluid loss results

#### 3.1.1 Non-composite mud fluid loss results

**Figure 11:**
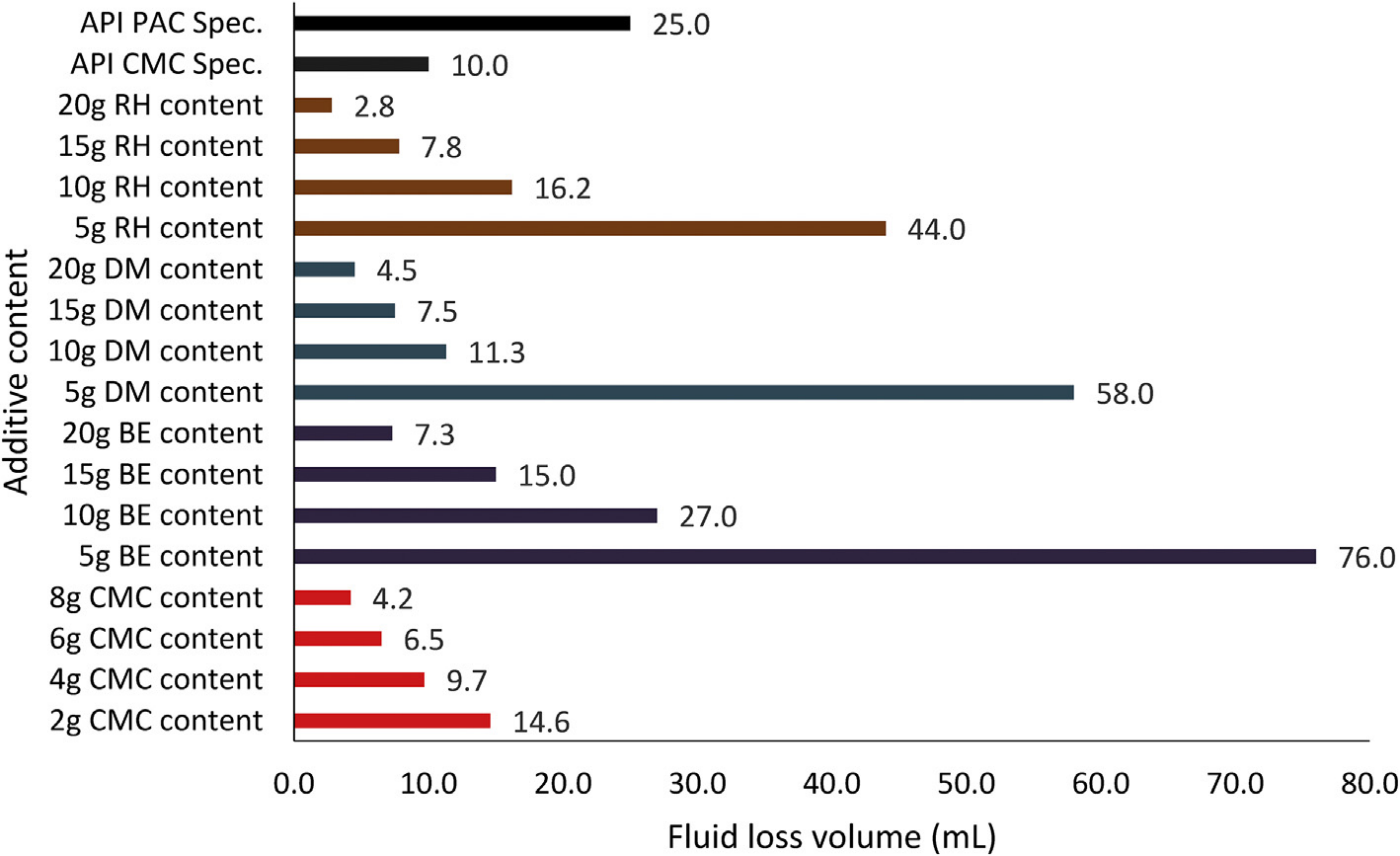
Filter loss volume for the drilling fluid (non-composite) samples at varying contents of the control additives.

**Figure 12:**
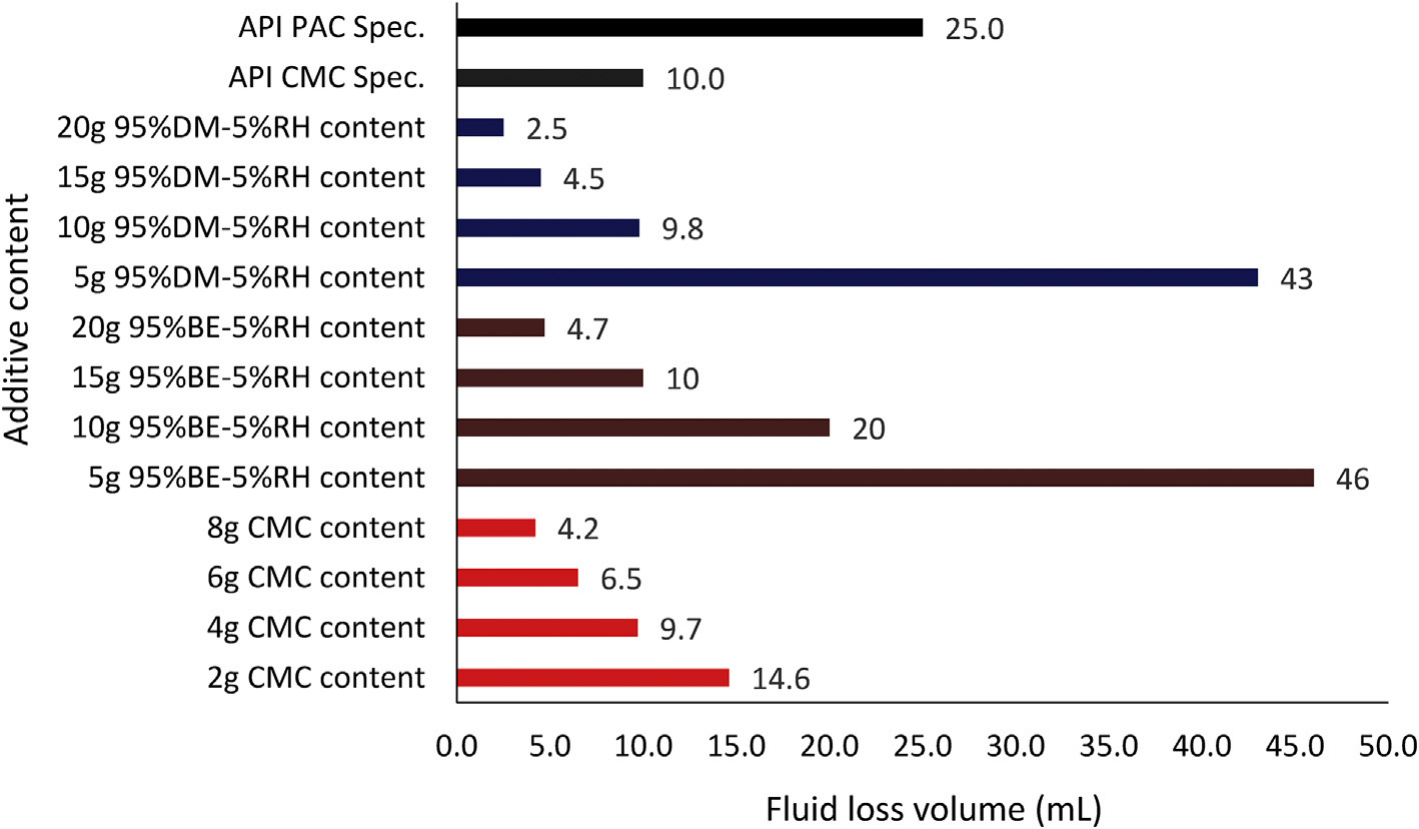
Filter loss volume for the drilling fluid samples at varying composite additive content (95% seed-5% rice husk).

**Figure 13:**
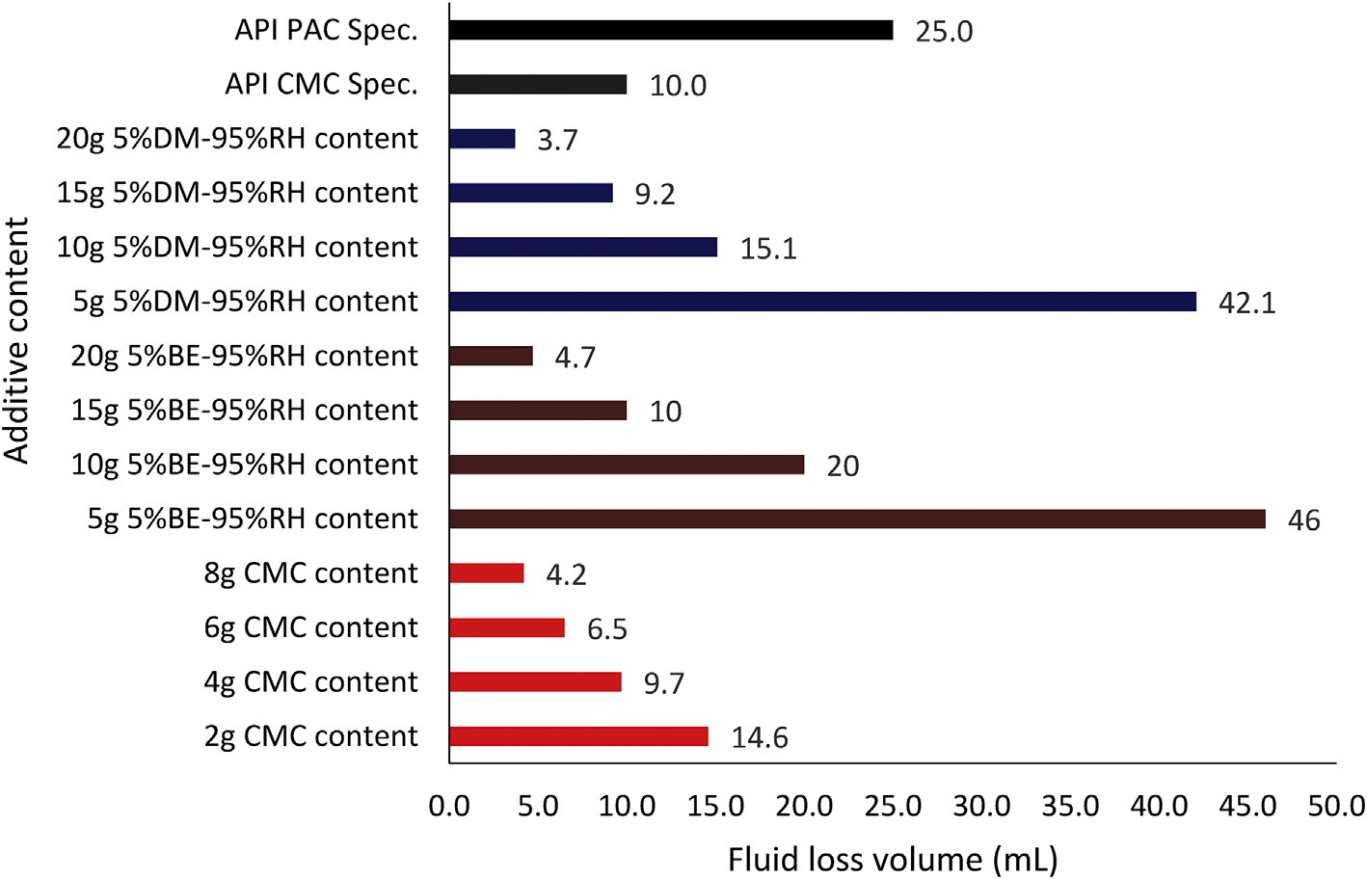
Filter loss volume for the drilling fluid samples at varying composite additive content (5% seed-95% rice husk).

Figures 11, 12 and 13 present the filter loss volume results obtained from the drilling fluid samples. As observed in Figure 11, it is clear that the local materials as fluid loss control additives (at low content) in water-based drilling fluid could not compete favourably with CMC in the water-based drilling fluid. However, an increase in the content of the local additives in the drilling fluid samples indicated that there was a significant decrease in the filter loss volume results obtained from the mud samples. This result is attributed to the increase in cellulose content in the drilling fluid as the additive content increases (Agwu et al., 2019). The results as presented in Figure 11 depict that rice husk and *Detarium microcarpum* at 10g content was comparable with 2g CMC content in the drilling fluid sample, while 15g *Brachystegia eurycoma* content was comparable with the same CMC content. Also, the results indicated that rice husk and *Detarium microcarpum* at 15g and 20g content had less fluid loss volume than CMC at 4g and 8g content, as *Brachystegia eurycoma* at 20g content could only have less fluid loss than 4g CMC content in the water-based drilling mud. Furthermore, comparing the filter loss performance of the local materials with API CMC and PAC specifications, this shows that the additives at 10g–20g content would perform favourably as CMC and PAC in the water-based drilling fluid. Thus, it can be said that the local fluid loss control additives have the potential to compare with CMC and PAC.

#### 3.1.2 Composite mud fluid loss results

The filter loss volume results for the composite drilling fluid samples are presented in Figures 12 and 13. Figure 12 depicts the results from the combination of 95% *Detarium microcarpum* and 5% rice husk (MUD-A), and 95% *Brachystegia eurycoma* and 5% rice husk (MUD-B) contents as fluid loss control additive in the drilling fluid samples. Figure 13 presents results obtained from 5% *Detarium microcarpum* and 95% rice husk (MUD-C), and 5% *Brachystegia eurycoma* and 95% rice husk (MUD-D) contents as fluid loss control additive in the mud samples. The results presented in Figure 8 showed that the additive (i.e., 95% *Detarium microcarpum* -5% rice husk) in MUD-A performed significantly to reduce the drilling mud filter loss volume than 95% *Brachystegia eurycoma*-5% rice husk control additive in MUD-B. Also, the results as presented in Figure 12 shows that the 95% *Detarium microcarpum*-5% rice husk additive in the water-based drilling mud will compare favourably with API CMC and more than API PAC specifications from additive content of 10g. For the 95% *Brachystegia eurycoma*-5% rice husk content in the drilling mud sample (MUD-B), it will perform close to the API CMC specification from additive content of 15g and more than API PAC specification from additive content of 10g. The fluid loss results obtained further showed that 10g of the composite additive in MUD-A was comparable with 4g of CMC content, while 15g and 20g composite additive (MUD-A) resulted in less fluid loss than 6g and 8g CMC content in the drilling fluid. For composite additive in MUD-B, it is observed that only 20g content was near the fluid loss volume obtained for 8g CMC content in the drilling mud. On the other hand, Figure 13 showed that the results (fluid loss volume) for the composite muds: MUD-C and MUD-D at 15g additive content can be compared to 4g CMC content. Furthermore, at 20g composite content, MUD-C fluid loss obtained was less than that of 8g CMC mud, while that of MUD-D was relatively close to the CMC mud at 8g content. Additionally, it is observed from Figure 13 that at additive content of 15g both 5% *Detarium microcarpum*-95% rice husk (MUD-C), and 5% *Brachystegia eurycoma*-95% rice (MUD-D) as filter loss control additive will perform remarkable than API PAC specification and compare favourably with API CMC specification. This result implies that these composite additives at the mentioned content would be capable to control fluid loss in water-based drilling fluid as the CMC and PAC.

#### 3.1.3 Comparison of composite and non-composite mud fluid loss resul

**Figure 14:**
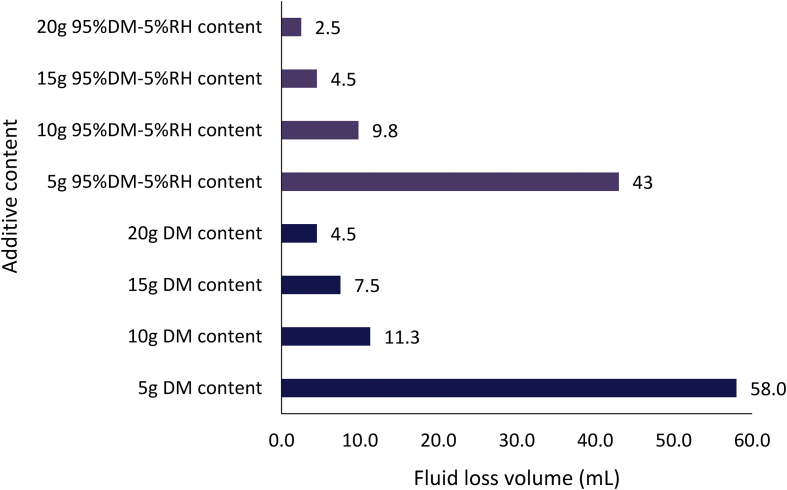
Comparison of the non-composite and composite mud filter loss volume at varying additive (*Detarium microcarpum*) content.

**Figure 15:**
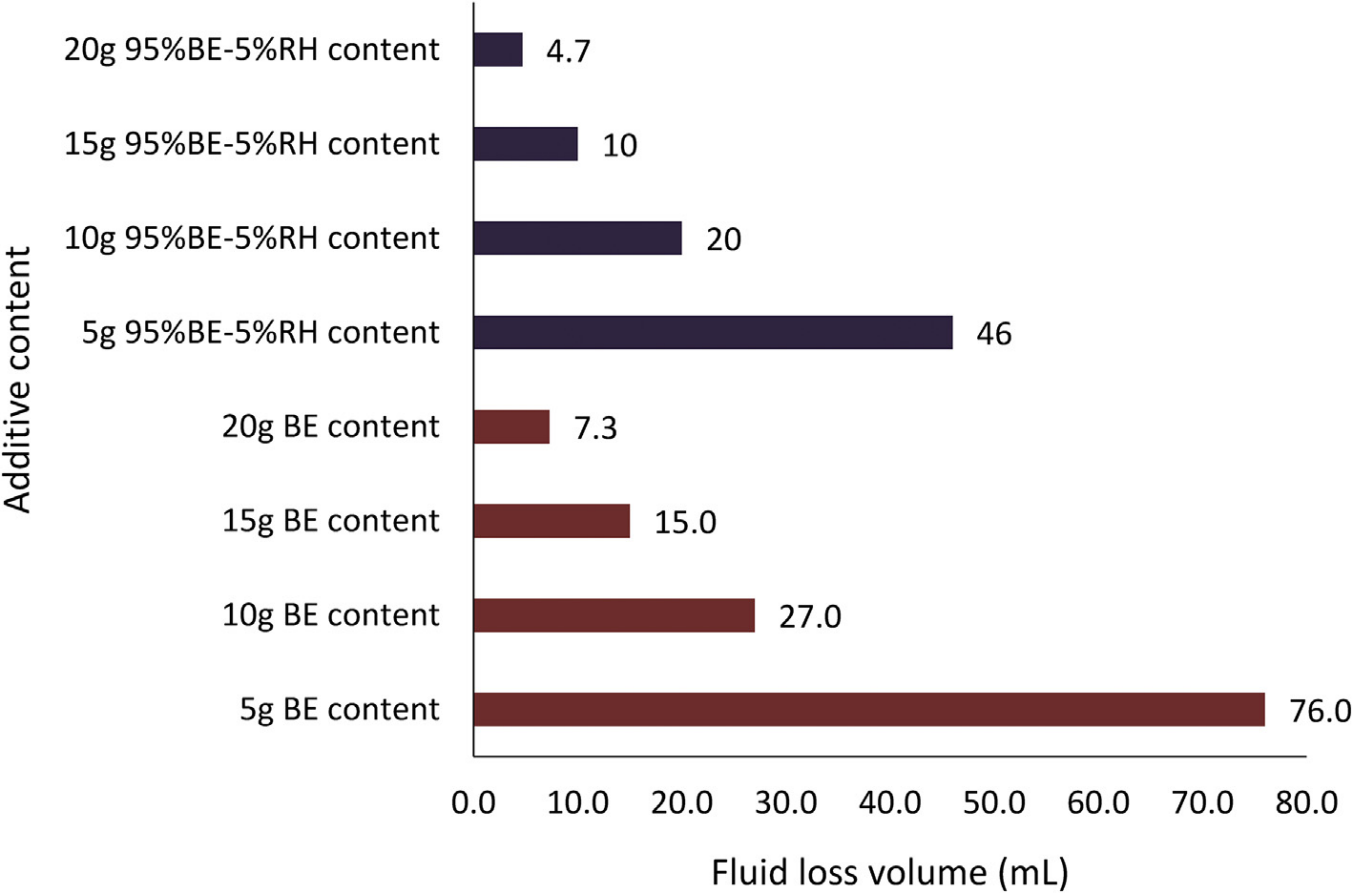
Comparison of the non-composite and composite mud filter loss volume at varying additive (*Brachystegia eurycoma*) content.

**Figure 16:**
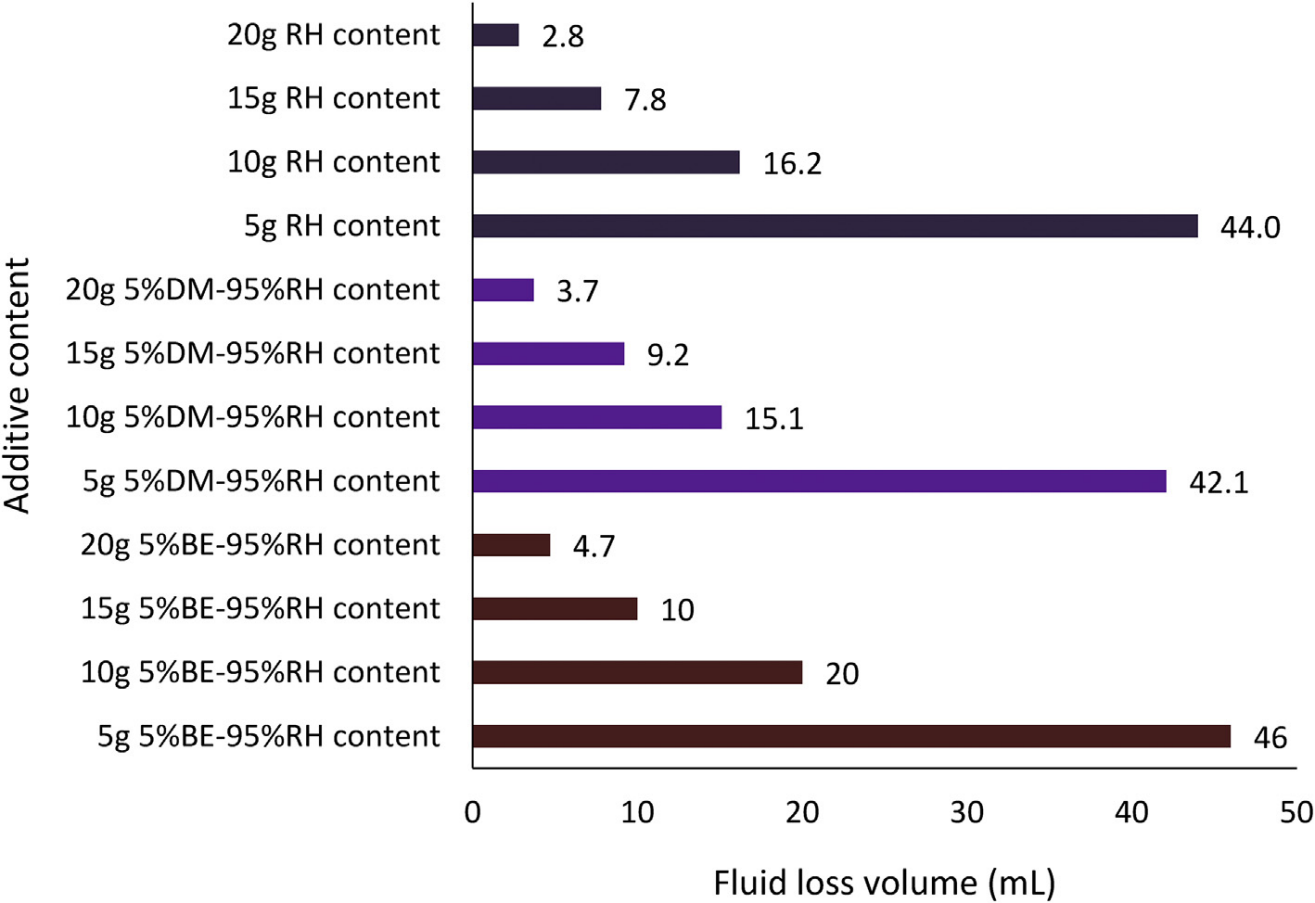
Comparison of the non-composite and composite muds filter loss volume at varying additive (rice husk) content.

Figures 14, 15 and 16 present the filter loss volume results for the non-composite and composite muds. For the *Detarium microcarpum* and *Brachystegia eurycoma* muds (i.e., non-composite muds), there was a noticeable decrease in the filter loss volume from their composite counterpart, that is MUD-A (95% *Detarium microcarpum*-5% rice husk) and MUD-B (95% *Brachystegia eurycoma*-5% rice husk) when compared to *Detarium microcarpum* and *Brachystegia eurycoma* muds (Figures 14 and 15). This result is because of the increase in cellulose content (from rice husk) in the composite muds than in its non-composite counterpart. Additionally, the Figures indicated that *Detarium microcarpum* composite mud (MUD-A) performed better than *Brachystegia eurycoma* composite (MUD-B) and was more comparable with the CMC mud as well as API specification for fluid loss control additive from 10g content. Further analysis of the results at 20g additive content showed composite additive (MUD-A) reduce fluid loss volume by 44.44% when compared to its non-composite complement (*Detarium microcarpum* mud). Also, composite additive MUD-B decreases the filter loss volume by 35.62% than its non-composite type (*Brachystegia eurycoma* mud). For the rice husk mud (non-composite) and its composite muds: MUD-C (5% *Detarium microcarpum*-95% rice husk) and MUD-D (5% *Brachystegia eurycoma* -95% rice husk), it is observed in Figure 16 that the rice husk filter loss control potential was slightly greater than that of its composite form. Thus, rice husk mud experienced increase fluid loss volume of 32.14% and 67.86% with composite MUD-C and MUD-D, respectively, at additive content of 20g. This result implied that adding *Detarium microcarpum* or *Brachystegia eurycoma* to the rice husk reduces its fluid loss control capability.

### 3.2 Filter cake results

#### 3.2.1 Filter cake thickness

##### 3.2.1.1 Non-composite mud

**Figure 17:**
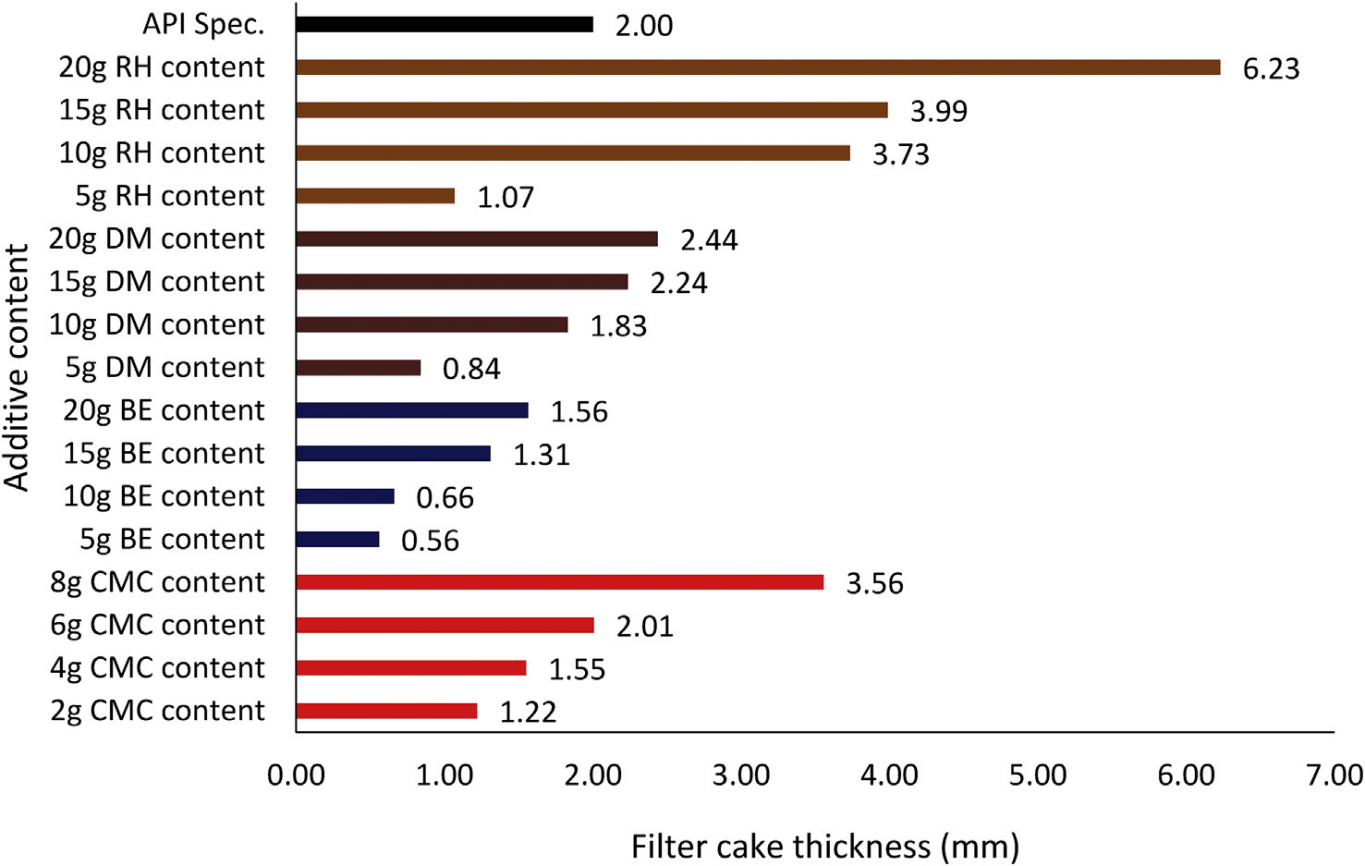
Filter cake thicknesses for the drilling fluid (non-composite) samples at varying contents of the control additives.

Figure 17 presents the filter cake thickness of the non-composite muds at different control additive contents. From the Figure, it is noted that all the non-composite mud filter cake thickness exhibited the same trend, that is, the filter cake thickness increases with increase in the additive content in the drilling mud samples. Comparing the filter cake thickness results showed that rice husk at 10g content was comparable with 8g CMC content as fluid control additive in the water-based mud. A close look at the Figure indicated that *Detarium microcarpum* mud cake thickness at 15g additive content was relatively the same as that of 6g CMC content, while *Brachystegia eurycoma* 20g filter performance was equivalent to 4g CMC content in the drilling fluid. Also, the filter cake thickness obtained from rice husk and *Detarium microcarpum* at the 10g and 15g content respectively met the API specification (Table 5) for filter cake thickness of fluid loss control additive. Furthermore, the results from the various non-composite muds' filter loss volume (Figure 11) and the filter cake thickness (Figure 17) showed that the muds’ filter loss volume decreased as the filter cake thickness increases.

##### 3.2.1.2 Composite mud

**Figure 18:**
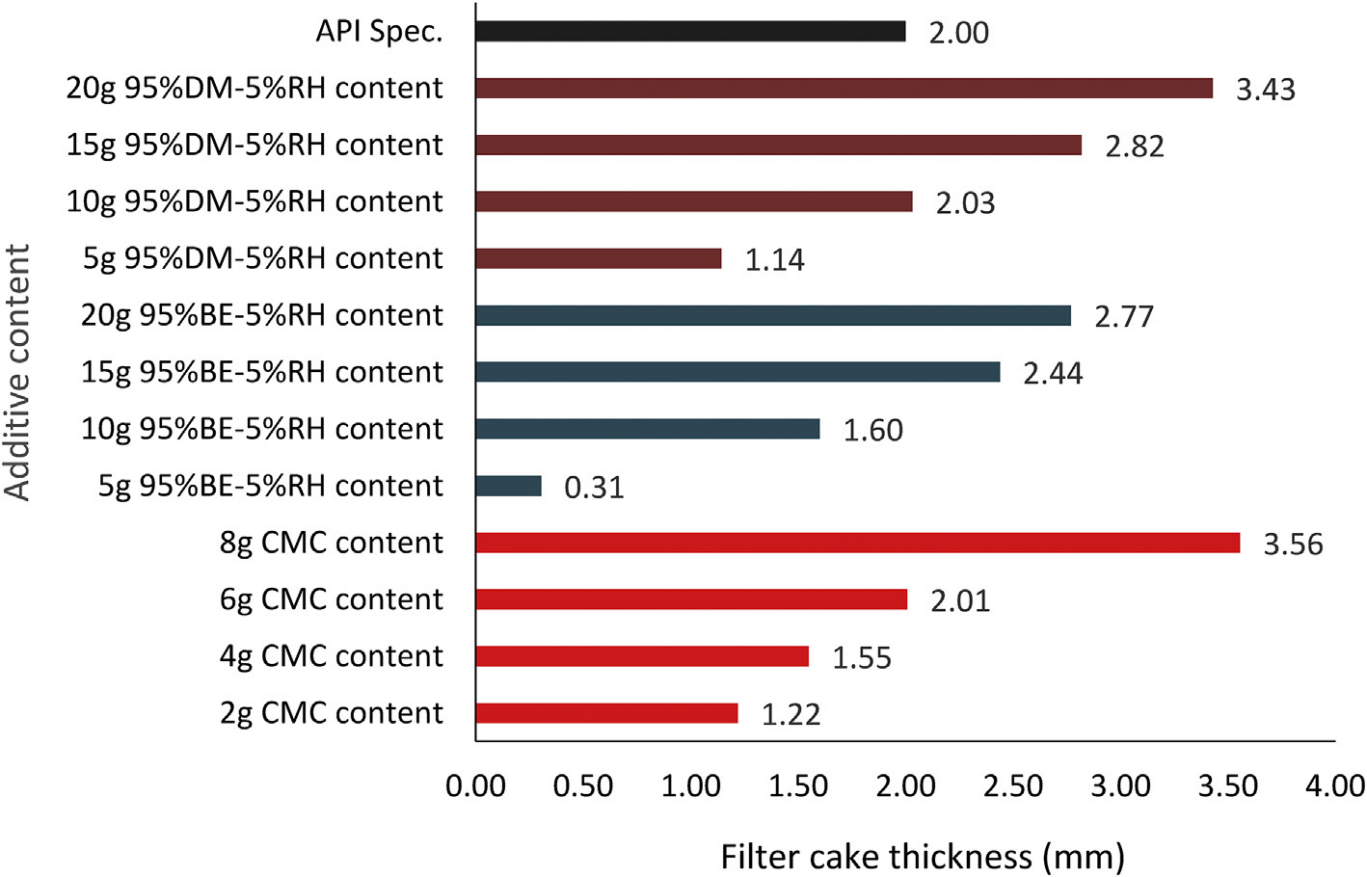
Filter cake thicknesses for the composite (*Detarium microcarpum*. and *Brachystegia eurycoma*) mud samples at varying contents of the control additives.

**Figure 19:**
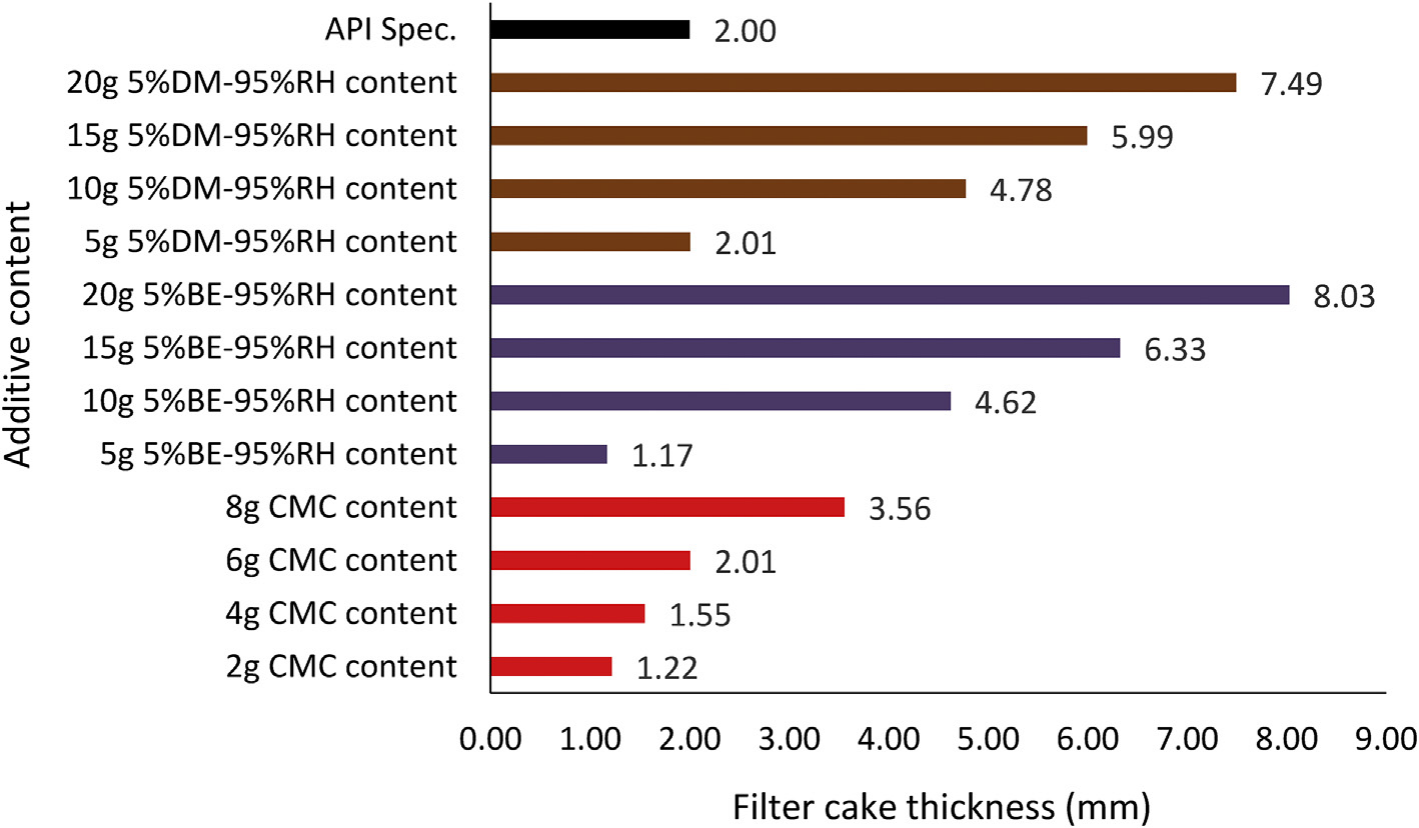
Filter cake thicknesses for the composite (rice husk) mud samples at varying contents of the control additives.

Figures 18 and 19 present the composite mud filter cake thickness results obtained for the various mud samples at different fluid loss control additive contents. The results obtained from the Figures followed the earlier mentioned trend. From Figure 18, it is noted that MUD-A (95% *Detarium microcarpum*-5% rice husk) filter thickness at additive content of 5g and 20g were closer to the CMC mud results at 2g and 8g content, while 10g and 15g MUD-A filter cakes were thicker than CMC mud cakes of 4g and 6g. Again, the result showed that 10g additive in MUD-A resulted in the same mud cake thickness with 6g CMC content in the drilling fluid sample. On the other hand, the mud cake thickness for composite MUD-B (95% *Brachystegia eurycoma*-5% rice husk) at additive content of 10g is comparable with 4g CMC mud. Also, it is noted that the filter cake thickness from MUD-B at additive content of 15g and 20g were thinner than 8g CMC content in the water-based mud. Furthermore, it is observed from the Figure that the filter cake thickness obtained for 20g MUD-B is close to 15g MUD-A. This observation means that composite MUD-A is more favourable than composite MUD-B when compared with CMC as a filtration control additive in the water-based drilling fluid.

Figure 19 indicated that composite MUD-C (5% *Detarium microcarpum*-95% rice husk) filter cake thickness at 5g additive content was the same as that of 6g CMC content in the drilling mud sample. Also, the results obtained for MUD-C and MUD-D (5% *Brachystegia eurycoma*-95% rice husk) at 10g additive content is relatively close. This is because the predominant additive material in the drilling fluid samples is rice husk, which is at the same quantity (or percentage, 95%). Hence, the Figure revealed that the filter cake thickness of the composite muds MUD-C and MUD-D outperformed (i.e., thicker than) the CMC mud at their equivalent comparable additive content (as presented in Table 4), except for MUD-D at 5g additive content. Again, comparing the various composite muds filter cake thickness results with the API specification for fluid loss control additive showed that MUD-A and MUD-D met the API specification at additive content of 10g, while MUD-B did so at 15g content, and MUD-C met the specification at 5g additive content. It is worthy to note here that, the increase in the filter cake thickness of the various composite mud samples implies a decrease in the filter loss volume of the composite muds as observed in Figures 12 and 13.

##### 3.2.1.3 Comparison of composite and non-composite mud

**Figure 20:**
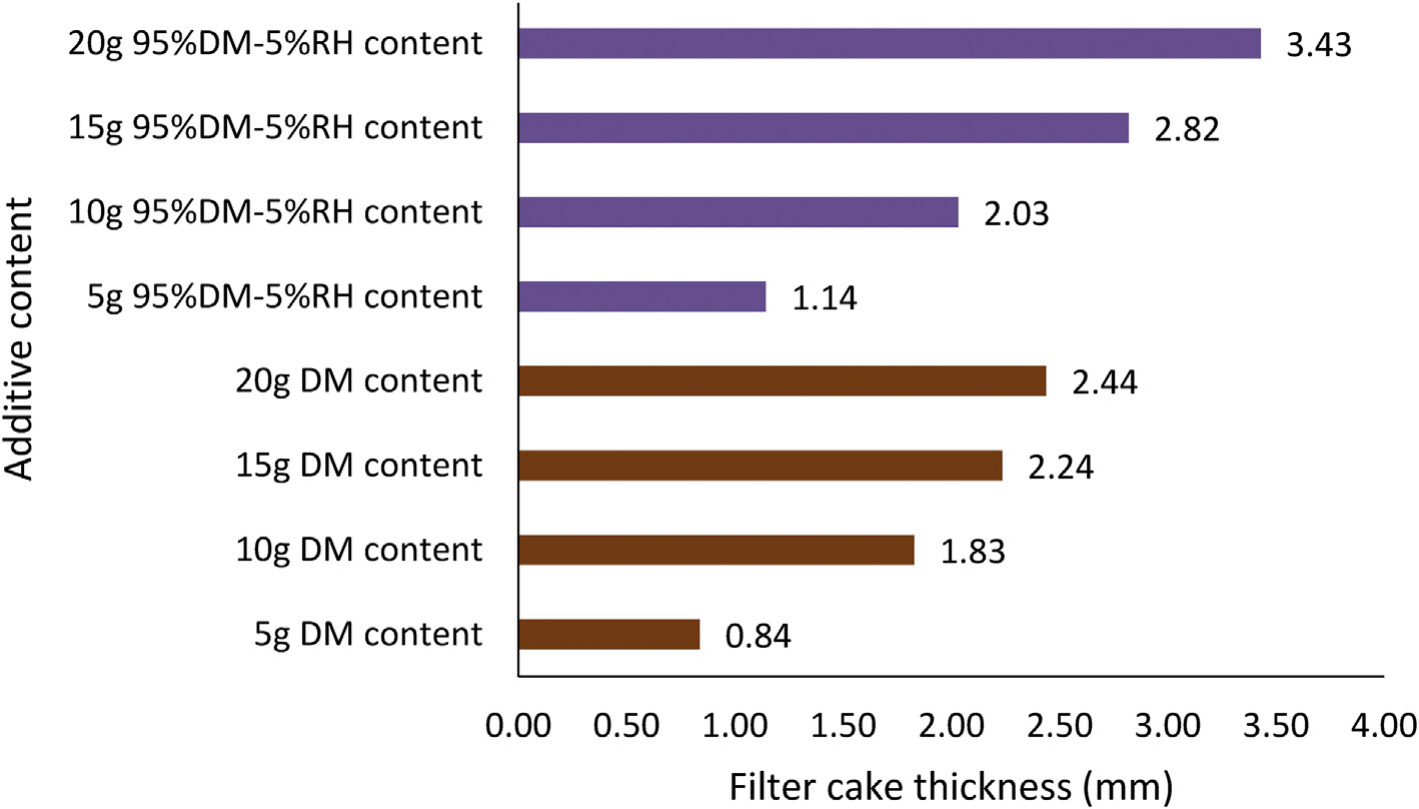
Comparison of the non-composite and composite muds filter cake thicknesses at varying additive (*Detarium microcarpum*) content.

**Figure 21:**
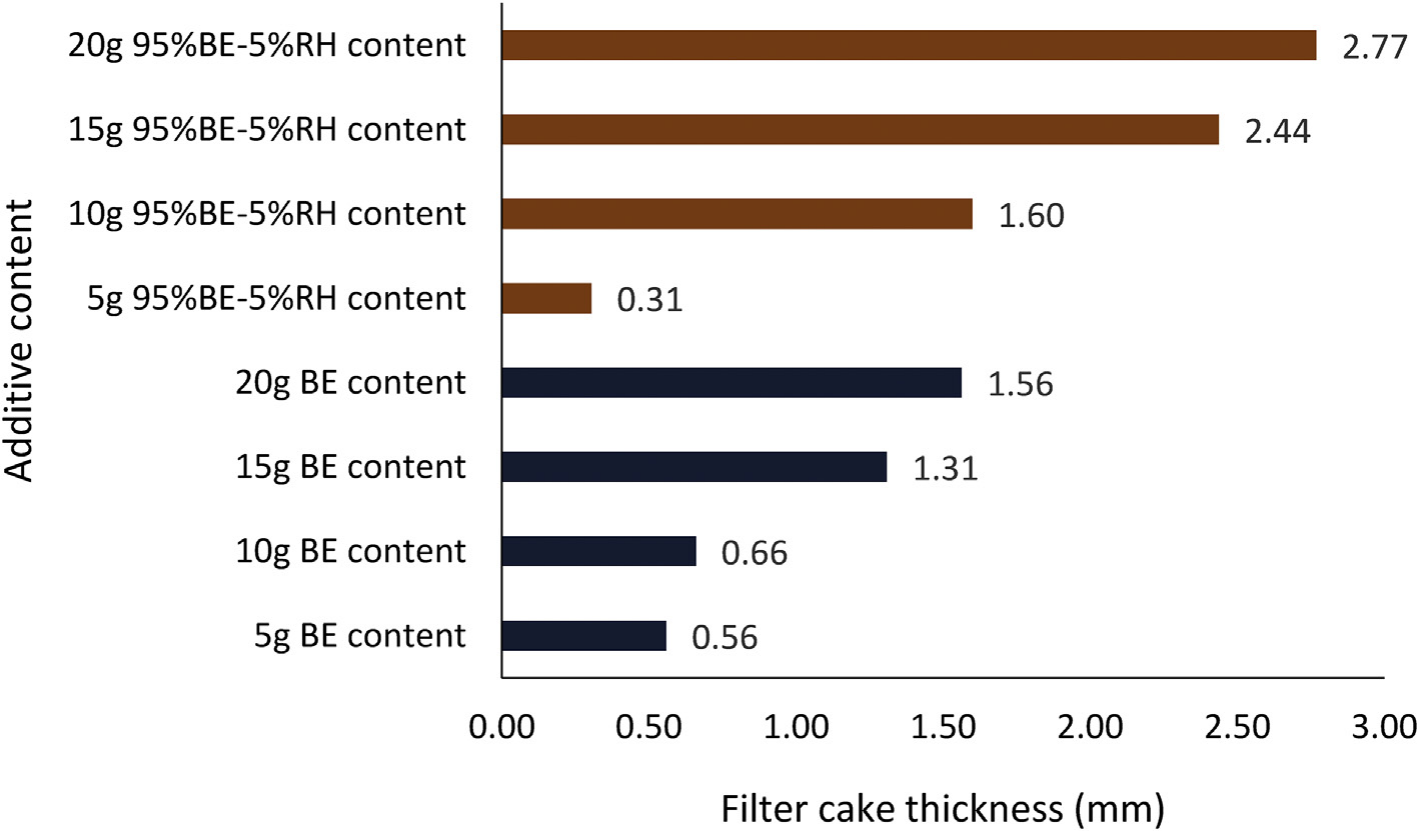
Comparison of the non-composite and composite muds filter cake thicknesses at varying additive (*Brachystegia eurycoma*) content.

**Figure 22:**
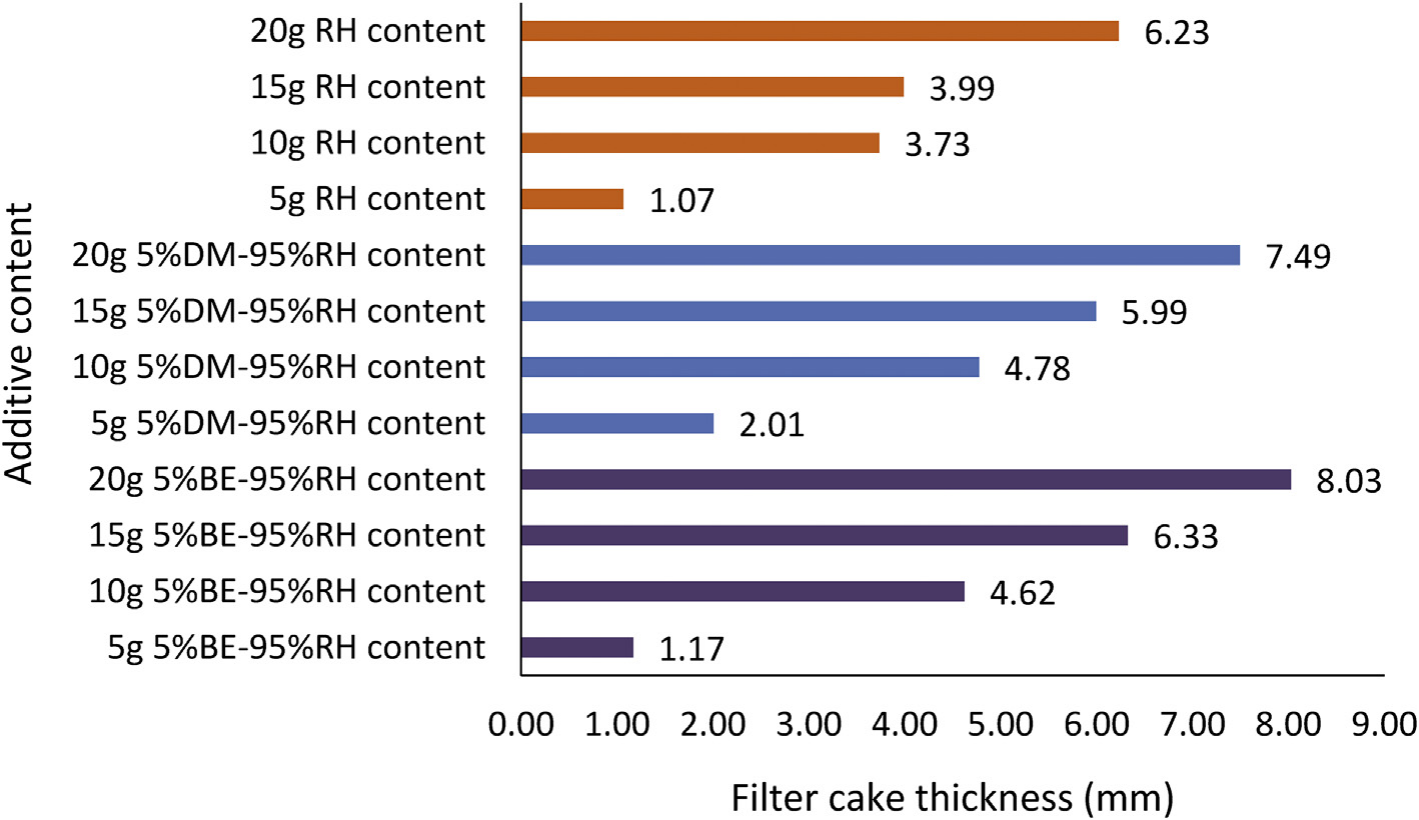
Comparison of the non-composite and composite muds filter cake thicknesses at varying additive (rice husk) content.

Figures 20, 21 and 22 depict the comparison of the non-composite and composite mud filter cake thickness results. A look at the Figures showed that thicker filter cakes were obtained from the composite muds: MUD-A and MUD-B than their corresponding non-composite muds: *Detarium microcarpum* and *Brachystegia eurycoma* (Figures 20 and 21). This performance of the composite muds: MUD-A and MUD-B are evidenced in the filter loss volume obtained (Figures 14 and 15). In Figure 21, the result further revealed that 10g composite additive (MUD-B) resulted in relatively the same filter cake thickness with the non-composite additive at 20g content in the drilling mud sample. In similarity with the observation in the composite muds MUD-A and MUD-B, in Figure 22 rice husk composite muds’ (i.e., MUD-C and MUD-D) filter cake thickness was thicker than its non-composite counterpart, that is, rice husk mud. This result implies that the combination of rice husk with either *Detarium microcarpum* or *Brachystegia eurycoma* as a fluid loss control additive in water-based drilling mud is preferable than rice husk for the same purpose in water-based mud. Also, the results in Figures 20, 21 and 22 indicated that the composite muds: MUD-C at all additive content, MUD-A and MUD-D from 10g content, and MUD-B from 15g, filter cake thicknesses were up to API specification of great than 2mm. Thus, these results further supported the earlier assertion that the composite additives are preferable than non-composite, which should be to be exploited. In all, the filter cake thicknesses of the composite and non-composite muds at 20g additive content showed that the composite muds MUD-A, MUD-B, MUD-C and MUD-D increased by 40.57%, 77.56%, 20.23% and 28.89%, respectively over their corresponding non-composite counterparts.

#### 3.2.2 Mud filter cake characteristics

**Figure 23:**
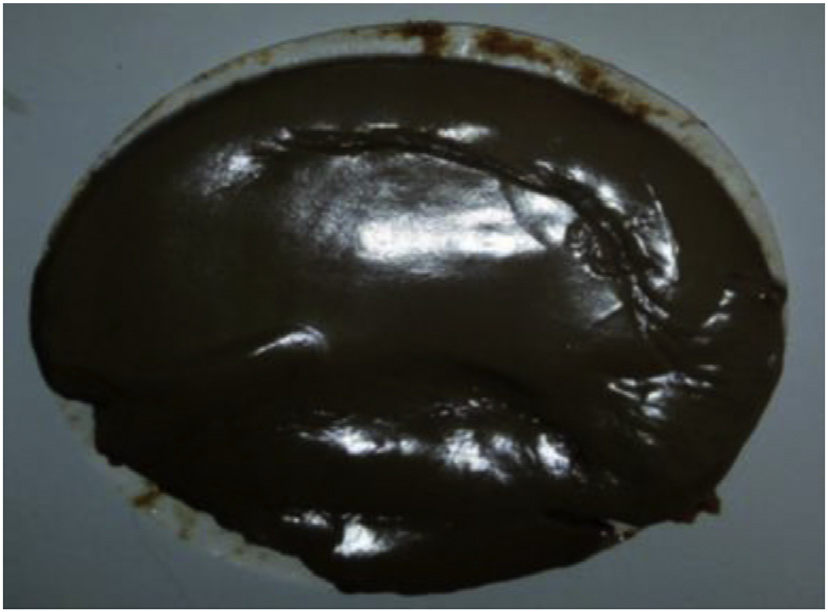
Rice husk mud filter cake.

**Figure 24:**
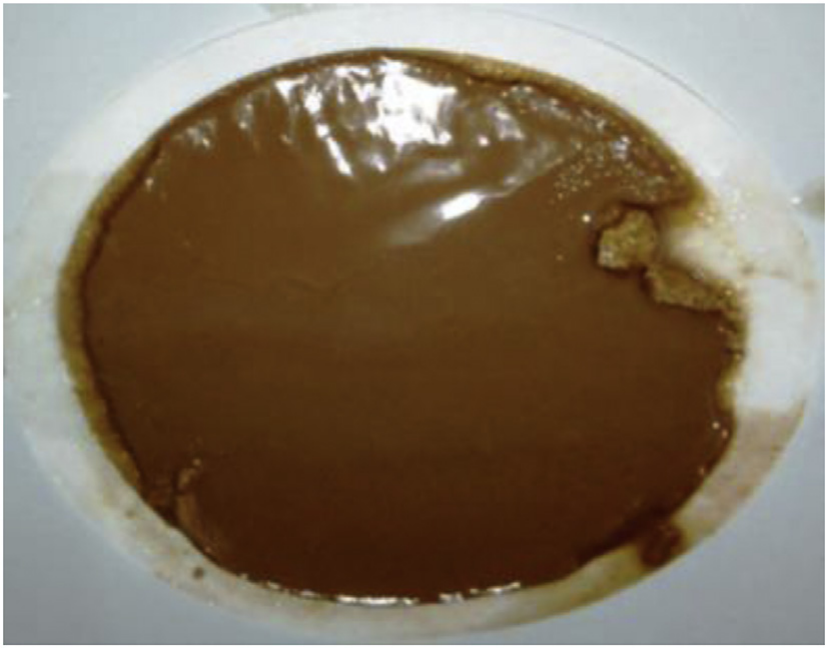
*Detarium microcarpum* mud filter cake.

**Figure 25:**
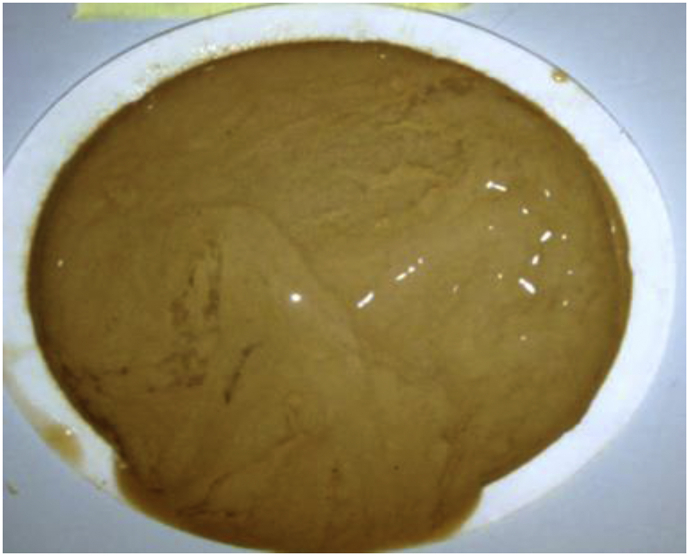
*Brachystegia eurycoma* mud filter cake.

**Figure 26:**
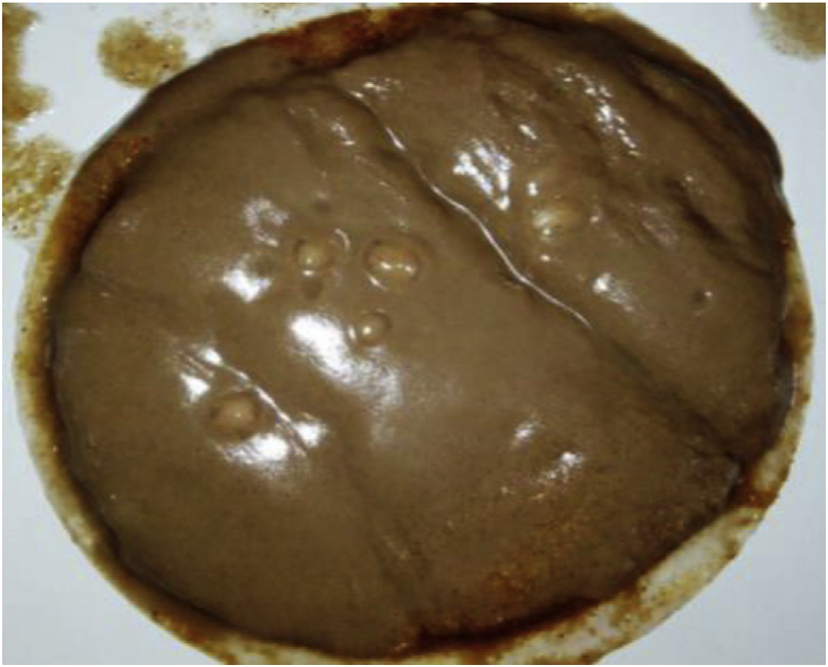
Composite (*Detarium microcarpum* - rice husk) mud filter cake.

**Figure 27:**
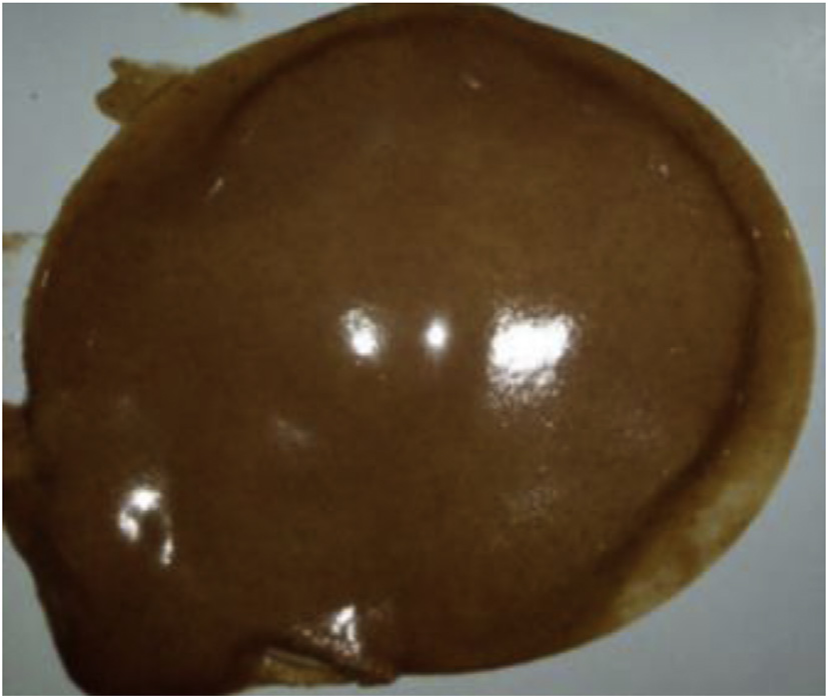
Composite (*Brachystegia eurycoma* - rice husk) mud filter cake.

Agwu et al. (2019) opined that there is no direct approach to evaluate the texture of a mud cake documented in the literature. Hence, researchers are left with subjective judgment to assess the mud cake texture. Therefore, physical evaluation of the various mud filter cakes; as presented in Figures 23, 24, 25, 26 and 27 was used in this study. Figures 23, 24 and 25 present the mud filter cakes obtained for the non-composite muds. Assessment of these mud cakes showed that their texture was slippery, smooth and soft. Also, these mud filter cake characteristics were observed for the composite muds, as seen in Figures 26 and 27. Thus, it is eminent to state here that the smooth and slippery characteristics of the muds are requirements of a good mud filter cake. This is because these characteristics of the mud cake prevent differential pipe sticking due to its sticky nature when compared to mud cake that is near dry and solid (Agwu et al., 2019). In addition, the soft and slippery characteristics of the mud filter cakes are preferable than solid and sticky texture, as the later mud characteristic will result in excessive torque and frictional drag when the drill pipe comes in contact with the wellbore walls (Anawe-Paul and Adewale, 2018; Agwu et al., 2019). In other words, the locally sourced materials exhibited good mud filter cake characteristics.

#### 3.2.3 Mud cake permeability

From the mud cake permeability results obtained for the various drilling mud samples at average mud viscosity of 1.12cP and 1.75cP for non-composite and composite muds, respectively (Table 6), it is observed that the mud cake permeability values decreased as the additive content in the mud sample increases. This observation explains why high filter loss volume and less filter cake thickness values were obtained at less additive content in the drilling fluid. From the results obtained, for static filtration test, it is pertinent to establish fluid loss control additive content - mud cake permeability outcome on filter (fluid) loss volume and filter cake thickness (Table 7), as this will present filtration parameters - control additive content relation in water-based mud.

**Table 6:** Mud cake permeability results.

Additive content (g)	Mud Cake Permeability (10^−3^mD)						
	Non-composite Mud			Composite Mud			
	DM mud	BE mud	RH mud	MUD-A	MUD-B	MUD-C	MUD-D
5	4.87	7.98	6.06	4.93	8.47	6.29	9.27
10	2.07	4.26	4.71	2.00	7.23	3.71	6.34
15	1.68	4.07	3.12	1.27	5.53	1.50	5.39
20	1.10	1.79	1.75	0.86	2.78	1.33	3.78

**Table 7:** Control additive content and fluid loss tests outcome.

	Control additive content	Mud cake permeability	Filter (fluid) loss volume	Filter cake thickness
i.	Less additive content	High value	High filter loss volume	Thin filter cake thickness
ii.	High additive content	Low value	Less filter loss volume	Thick filter cake thickness

##### 3.2.3.1 Non-composite mud cake permeability

**Figure 28:**
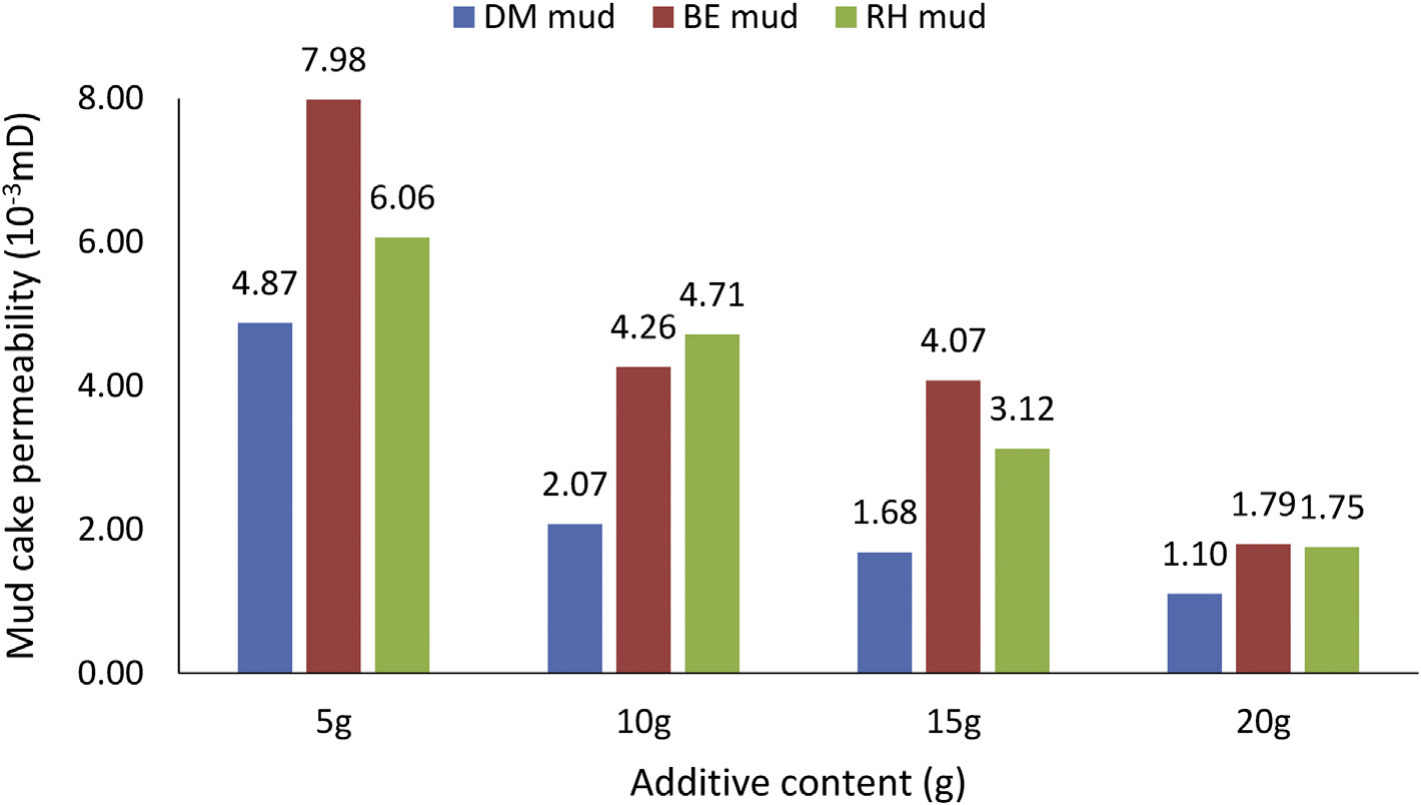
Mud cake permeability of non-composite muds at different control additive contents.

The comparison of the non-composite muds’ cake permeability results obtained (Figure 28) indicated that all the muds: *Detarium microcarpum*, *Brachystegia eurycoma* and rice husk, exhibits high cake permeability at 5g content and less at 20g content in the water-based drilling mud. This result means that the mud cake permeability of the non-composite muds decreases as the fluid loss control additive content in the drilling mud increases. Also, the Figure presented shows that *Brachystegia eurycoma* mud has higher mud cake permeability at different additive content than others. This result explains the reason for the high filter loss and thin filter cake obtained from *Brachystegia eurycoma* as fluid loss control additive in the water-based mud when compared to *Detarium microcarpum* and rice husk muds; as indicated in Figure 11.

##### 3.2.3.2 Composite mud cake permeability

**Figure 29:**
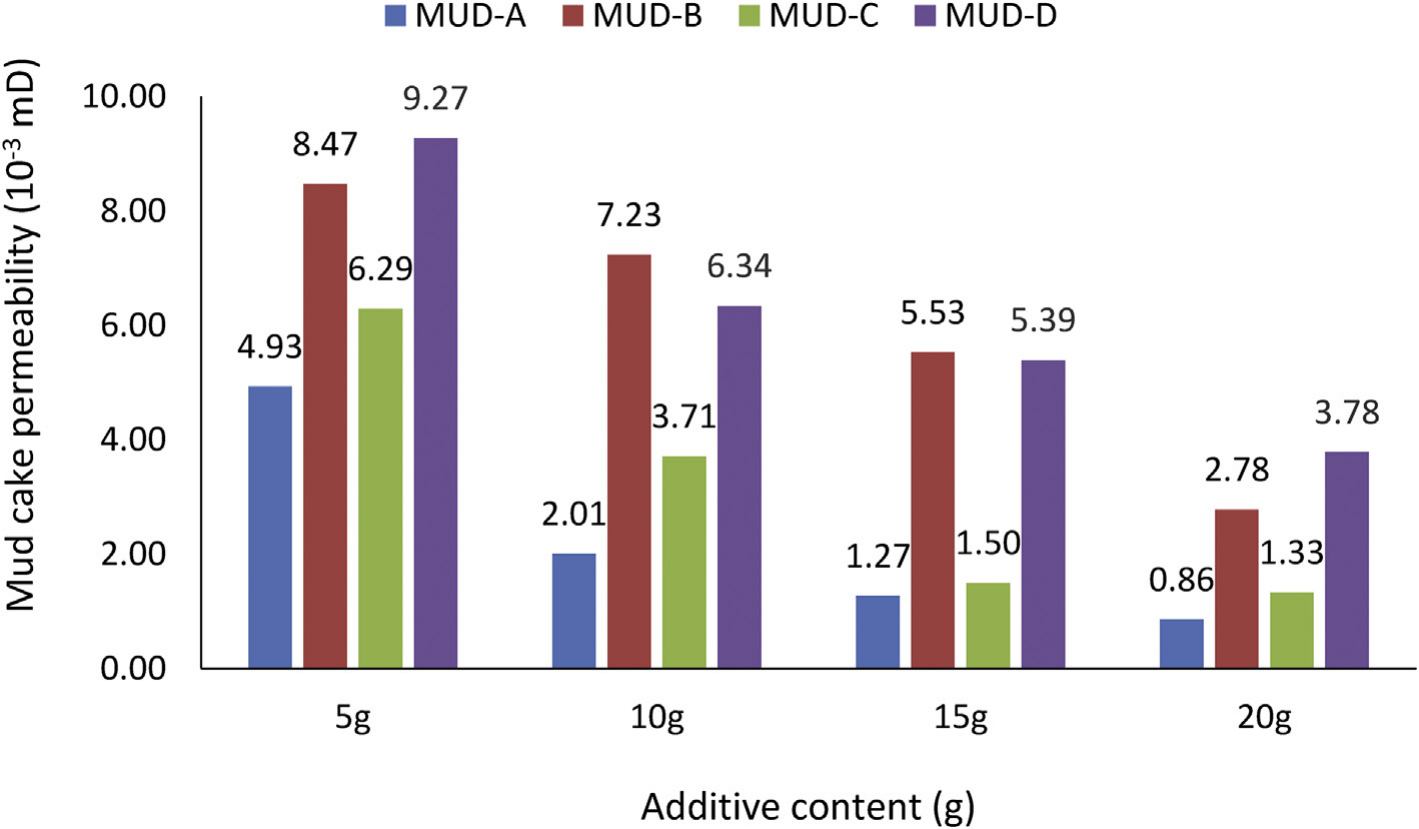
Mud cake permeability of composite muds at different control additive contents.

Figure 29 presents the mud cake permeability results obtained for the various composite muds (i.e., MUD-A through MUD-D) at different additive content. As already established, the mud cake permeability obtained for the composite muds decreased as the fluid loss control additive content increased in the mud sample. This is in line with the established fluid loss control additive content – mud cake permeability outcome for static filtration test (Table 7). From the Figure; it is observed that MUD-A (i.e., 95% *Detarium microcarpum*-5% rice husk mud) has the least mud cake permeability at the different additive content in the mud samples. This observation explains the reason for the favourable and comparable performance of the composite additive (i.e., 95% *Detarium microcarpum*-5% rice husk) as fluid control in the water-based mud with CMC and PAC, as presented in Figure 12.

##### 3.2.3.3 Comparison of non-composite and composite muds cake permeability

**Figure 30:**
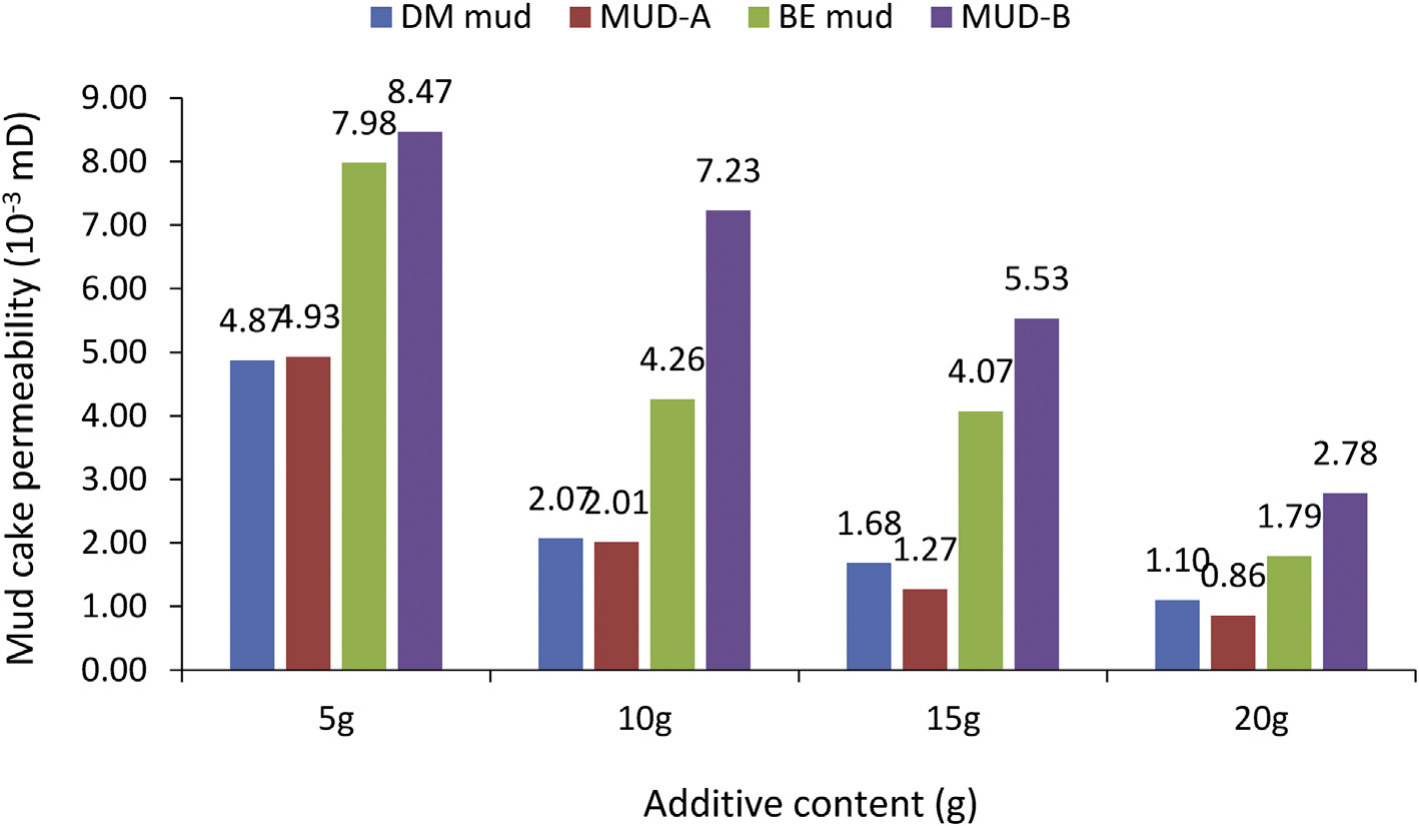
Comparison of the non-composite and composite muds cake permeability at different contents (*Detarium microcarpum* and *Brachystegia eurycoma*).

From the results obtained, in Figure 30, it is clear that high mud cake permeability values were obtained for the composite MUD-B mud (i.e., 95% *Brachystegia eurycoma*-5% rice husk) than in non-composite *Brachystegia eurycoma* mud. Also, for the composite *Detarium microcarpum* mud and its non-composite counterpart (95% *Detarium microcarpum*-5% rice husk) mud, have about the same mud cake permeability values. This is because there was no much difference between these muds’ cake thickness values; as presented in Figure 20. As stated in Table 7, the results obtained showed that the cake permeability of these muds decreased as the additive content in the muds increases. The reason for this observation is because increases in additive content in the mud improve the filtration control characteristic of the drilling mud (Okon et al., 2014).

**Figure 31:**
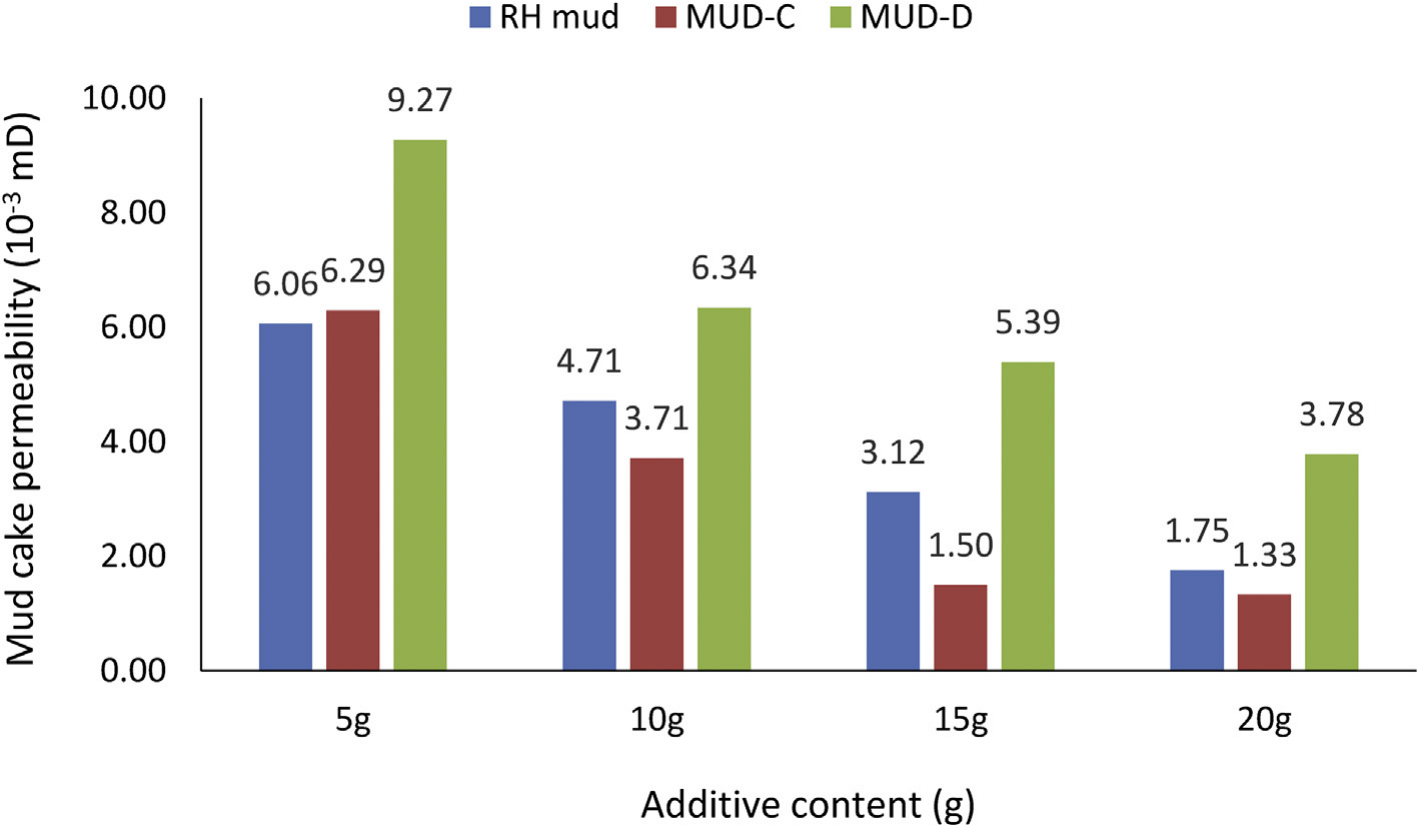
Comparison of the non-composite and composite muds cake permeability at different contents (rice husk).

In line with the observations in Figure 30, comparing rice husk (non-composite) mud with its composite complements: MUD-C and MUD-D (i.e., 5% *Detarium microcarpum*-95% rice husk and 5% *Brachystegia eurycoma*-95% rice husk, respectively), the results obtained depicts that mud cake permeability values of the composite MUD-D were higher than that of non-composite mud (Figure 31). On the other hand, the mud cake permeability results for the composite MUD-C were lower than the non-composite rice husk mud. Exception to this was observed in 5g additive content, as the filter cake thickness obtained for the non-composite rice husk mud was low. Thus, the high mud cake permeability value of the composite mud (MUD-D) explains the reason for its less favourable fluid loss volume and filter cake thickness when compared with composite MUD-C. Again, the results also presented the aforementioned mud cake permeability - additive content trend in the water-based drilling fluid. Worth noting from the results, is that, low mud cake permeability imply efficient filter cake thickness, as observed from both composite and non-composite additive content in water-based drilling fluid.

## 4 Conclusion

For successful drilling operation, drilling fluid “the blood of the operation” is expected to perform critical functions. One of these functions is to seal the walls of the formation being drilled to prevent filtration. Several commercial fluid loss control additives, namely, CMC, PAC, among others had been in use with their limitations and financial implications on the overall drilling cost. In this study, several locally sourced materials: *Detarium microcarpum*, *Brachystegia eurycoma* and rice husk, were evaluated as fluid loss control additives in water based drilling fluid and the following conclusions can be drawn:

i. the locally sourced materials: *Detarium microcarpum* and rice husk at 15g content, and *Brachystegia eurycoma* at 20g content were comparable with 4g CMC fluid loss control potential in water based drilling fluid, and were within API specification for fluid loss control additives;
ii. in composite (combined) form, *Detarium microcarpum* - rice husk and *Brachystegia eurycoma* - rice husk at 10g and 15g additives, respectively, were similar to 4g CMC filtration loss control capability in water based drilling mud;
iii. at 20g content, composite additives: *Detarium microcarpum* - rice husk and *Brachystegia eurycoma* - rice husk reduce fluid loss by 44.44% and 35.62%, as filter cake increases by 40.57% and 77.56% over its non-composite counterpart;
iv. the mud cake characteristics of these locally sourced materials are acceptable for good mud filter cake requirement;
v. the mud cake permeability depends on fluid loss additive contents in the drilling mud, thus, low additive content results in high mud cake permeability; and
vi. fluid loss volume and filter cake thickness were mud cake permeability dependent, as high mud cake permeability gives high fluid loss volume and thin mud cake, while low mud cake permeability result in less fluid loss volume and thick mud cake.

It is recommended that further work be carried out on the locally sourced materials to establish their composite potential and/or effect on water-based drilling fluid viscosity (i.e., rheology parameters) at different thermodynamic conditions.

## Declarations

### Author contribution statement

Anietie N. Okon: Conceived and designed the experiments; Contributed reagents, materials, analysis tools or data; Wrote the paper.

Julius U. Akpabio: Analyzed and interpreted the data; Wrote the paper.

Kilaliba W. Tugwell: Performed the experiments.

### Funding statement

This research did not receive any specific grant from funding agencies in the public, commercial, or not-for-profit sectors.

### Competing interest statement

The authors declare no conflict of interest.

### Additional information

No additional information is available for this paper.

## References

[bib1] Abreu P., Relva A. (2002). Carbohydrates from *Detarium microcarpum* bark extract. Carbohydr. Res..

[bib2] Adebayo T.A., Chinonyere P. (2012). Sawdust as a filtration control and density additives in water-based drilling mud. Int. J. Sci. Eng. Res..

[bib3] Adebayo T.A., Osiwi A., Nwafor-Orizu E.N. (2012). Laboratory study effect of 1.0mm sawdust with various types of viscosifier additives on properties of oil-based mud. Int. J. Sci. Eng. Res..

[bib4] Agwu O.E., Akpabio J.U. (2018). Using agro-waste materials as possible filter loss control agents in drilling muds. J. Petrol. Sci. Eng..

[bib5] Agwu O.E., Akpabio J.U., Archibong G.W. (2019). Rice husk and saw dust as filter loss control agents for water-based muds. J. Heliyon.

[bib6] Akoko G.A., Ephriam M.E., Akobo I.Z.S., Ukpata J.O. (2012). Structural properties of rice husk ash concrete. Int. J. Eng. Appl. Sci..

[bib7] Amanullah M., Tan C.P. (2001). Embedment modulus of mudcakes - its drilling engineering significance. Paper Presented at the American Association of Drilling Engineers 2001 National Drilling Conference.

[bib8] Anawe-Paul A.I., Adewale F.J. (2018). Experimental investigation of fluid loss and cake thickness control ability of zirconium (IV) oxide (ZRO2) nanoparticles in water based drilling mud. Int. J. Eng. Technol..

[bib9] Anawe-Paul A.I., Efeovbokhan V.E., Adebayo T.A., Nwaogwugwu M.M. (2014). The effect of rice husk and sawdust on the properties of oil-based mud at varied temperatures. J. Energy Technol..

[bib10] Annudeep S.A. (2012). Rheological Properties and Corrosion Characteristics of Drilling Mud Additivies.

[bib55] Azizi A., Ibrahim M.S.N., Hamid K.H.K., Sauki A., Ghazali N.A., Mohd T.A.T. (2013). Agarwood Waste as a New Fluid Loss Control Agent in Water-Based Drilling Fluid.. Int. J. Sci. Eng..

[bib12] Azar J.J., Samuel G.R. (2007). Drilling Engineering.

[bib13] Bageri B.S., Al-Mutairi S.H., Mahmond M.A. (2013). Different techniques for characterizing the filter cake. Paper Presented at the Society of Petroleum Engineers Unconventional Gas Conference and Exhibition.

[bib14] Bazarnova N.G., Chubik P.S., Khmelnitskii A.G., Galochkin A.I., Markin V.I. (2001). Carboxymethylated wood as a chemical reagent for preparation of drilling fluids. Russ. J. Appl. Chem..

[bib15] Bourgoyne A.T., Millheim K.K., Chenevert M.E., Young F.S. (2003).

[bib16] Caenn R., Chillingar G.V. (1996). Drilling fluids: state of the art. J. Petrol. Sci. Eng..

[bib17] Chinwuba I.K., Princewill O.N., Vivian O.C. (2016). Evaluation of the fluid loss properties of pleurotus and its commercial availability. Int. J. App. Innov. Eng. Manag..

[bib18] Dagde K.K., Nmegbu C.G.J. (2014). Drilling fluid formulation using cellulose generated from groundnut husk. Int. J. Adv. Res. Tech..

[bib19] Davoodi S., Ahmed Ramazani S.A., Jamshidi S., Jahromi A.F. (2018). A novel field applicable mud formula with enhanced fluid loss properties in high pressure-high temperature well condition containing pistacho shell powder. J. Petrol. Sci. Eng..

[bib20] Egun I.L., Abah A.M. (2013). Comparative performance of cassava starch to PAC as fluid loss control agent in water based drilling mud. Discovery J..

[bib21] Esu C.O. (2016). Simulation and Optimization of Hydrogen Rich Syngas Production from the Stream Co-gasification of Coal and Rice Husk Using Aspen Plus.

[bib22] Feng Z., Hongming T., Yingfeng M., Gao L., Xijin X. (2009). Damage evaluation for water-based underbalance drilling in low permeability and tight sandstone gas reservoir. J. Petreo. Exp. Develop..

[bib23] Ghazali N.A., Mohd T.A.T., Alisa N.H., Azizi A., Harun A.A. (2014). The effect of lemongrass as lost circulation material (LCM) to the filtrate and filter cake formation. Key Eng. Mater..

[bib24] Hamida T., Kuru E., Pickard M. (2010). Filtration loss characteristics of aqueous waxy hull-less Barley (WHB) solutions. J. Petrol. Sci. Eng..

[bib25] Harry T.F., Joel O.F., Ademiluyi F.T., Oduola K. (2016). Performance evaluation of local cassava starches with imported starch for drilling fluid. Am. J. Energy Res..

[bib26] Herzraft B., Rousseau L., Neau L., Moan M., Bossard F. (2001). Influence of temperature and clay/emulsion microstructure on oil-based mud low shear rate rheology. Soc. Petrol. Eng. J..

[bib27] Hossain M.E., Wajheeuddin M. (2016). The use of grass as an environmentally friendly additive in water-based drilling fluids. J. Petrol. Sci..

[bib28] Ikegwu O.J., Okechukwu P.E., Ekumankana E.O. (2010). Physio-chemical and pasting characteristics of flour and starch from achi *Brachystegia eurycoma* seed. J. Food Technol..

[bib29] International Rice Research Institute (2016). Rice Knowledge Bank: Rice Husk.

[bib30] Iscan A.G., Kok M.V. (2007). Effects of walnut shells on the rheological properties of water-based drilling fluids. J. Energy Source A: Rec. Util. Environ. Effect.

[bib31] Ismail M.S., Waliuddin A.M. (1996). Effect of rice husk ash on high strength concrete. Construct. Build. Mater..

[bib32] Irondi E.A., Oboh G., Akindahunsi A.A. (2015). Methanol extracts of *Brachystegia eurycoma* and *Detarium microcarpum* seeds flours inhibit some key enzymes linked to the pathology and complications of type 2 diabetes in vitro. J. Food Sci. Human Wellness.

[bib33] Kosynkin D.V., Ceriotti G., Wilson K.C., Lomeda J.R., Scorsone J.T., Patel A.D., Friedheim J.E., Tour J.M. (2011). Graphene oxide as a high-performance fluid-loss-control additive in water-based drilling fluids. ACS App. Mater. Interface.

[bib34] Kumar A., Mohanta K., Kumar D., Parkash O. (2012). Properties and industrial applications of rice husk: a review. Int. J. Emerg. Technol. Adv. Eng..

[bib35] Lomba R. (2010). Fundamentos de Filtracao e Controle das Propriedades de Filtracao.

[bib36] McCosh K., Getliff J. (2004). Effect of drilling fluid components on composting and the consequences for mud formulation. Paper Presented at the American Association Drilling Engineers Drilling Fluids Conference.

[bib37] Nmegbu C.G.J., Bari-Agara B. (2014). Evaluation of corn cob cellulose and its suitability for drilling mud formulation. Int. J. Eng. Res. Afr..

[bib38] Nwakaudu A.A., Nwakaudu M.S., Owuamanam C.I., Alagbaoso S.O., Njoku N.E., Agunwah I.M., Ofoedu C., Ojukwu M., Ofoegbu J.C., Anikwenze R.O. (2017). Extraction and evaluation of hydrocolloids from “achi” (*Brachystegia eurycoma*) and its application on a water melon fruit juice. Eur. J. Food Sci. Technol..

[bib39] Nwosu J.U. (2012). The rheological and proximate properties of some food thickeners (“Ukpo”. “Achi” and “ofo”) as affected by processing. Int. J. Basic Appl. Sci..

[bib40] Nyeche W.E., Nmegbu C.G.J., Ifeoma P.J. (2015). Drilling mud formulation using potato starch (*Ipomoea batatas*). Int. J. Eng. Res. Afr..

[bib41] Odunukwe R.C. (2015). Suitability of *Detarium Microcarpum* as an Additive in Drilling Fluid. http://www.uniprojectsearch.com/suitability-detarium-microcarpum-additive-drilling-fluid/.

[bib42] Okon A.N., Udoh F.D., Bassey P.G. (2014). Evaluation of rice husk as fluid loss control additive in water based drilling mud. Paper Presented at the Society of Petroleum Engineers Nigerian Annual International Conference and Exhibition.

[bib43] Olatunde A.O., Usman M.A., Olafadehan O.A., Adeosun T.A., Ufot O.E. (2012). Improvement of rheological properties of drilling fluid using locally based materials. J. Petroleum Coal.

[bib44] Oleas A., Osuji C.E., Chenevert M.E., Sharma M.M. (2008). Entrance pressure of oil based mud into shale: effect of shale water activity and mud properties. Paper SPE 116364 Presented at Annual Technical Conference and Exhibition.

[bib45] Prasad R., Sankhyan S.K., Karim S.A. (2000). Utilization of different protein supplements in the diet of broiler rabbits. Indian J. Anim. Sci..

[bib46] Rautela M.S. (2000). A Method for Determination of the Permeability of the Filter Cake at Wellsite.

[bib47] Saengdee A., Terakulsatit B. (2017). Utilization of Sugarcane Bagasse Ash as Filtration Loss Control Agent in Water Based Drilling Muds.

[bib48] Samavati R., Abdullah N. (2016). The experimental assessment and study of ubi kayu starch as fluid loss control agent in water based drilling fluids. Int. J. Sci. Res. Chem. Eng..

[bib49] Subbukrishna A.S., Suresh T.E., Paul P.K., Dasappa M.K., Rajan T.R. (2007). Precipitated Silica from rice husk. Paper Presented at the 15th European Biomass Conference and Exhibition.

[bib50] Tugwell K.W. (2018). Evaluation of Locally Sourced Filter Loss Control Materials for Water Based Drilling Fluid.

[bib51] Udoh F.D., Okon A.N. (2012). Formulation of water-based drilling fluid using local materials. Asian J. Microbiol. Biotechnol. Environ. Sci..

[bib52] Udoh F.D., Itah J.J., Okon A.N. (2012). Formulation of synthetic-based drilling fluid using palm oil derived ester. Asian J. Microbiol. Biotechnol. Environ. Sci..

[bib53] Uhegbu F.O., Onwuchekwa C.C., Iweala E.E.J., Kanu L. (2009). Effect of processing methods on nutritive and antinutritive properties of seeds of *Brachystegia eurycoma* and *Detarium microcarpum* from Nigeria. Pakistan J. Nutr..

[bib54] Ummah H., Suriamihardja D.A., Selintung M., Wahab A.W. (2015). Analysis of chemical composition of rice husk used as absorber plates sea water into clean water. ARJN J. Eng. App. Sci..

